# *SLC20A1* Is Involved in Urinary Tract and Urorectal Development

**DOI:** 10.3389/fcell.2020.00567

**Published:** 2020-08-07

**Authors:** Johanna Magdalena Rieke, Rong Zhang, Doreen Braun, Öznur Yilmaz, Anna S. Japp, Filipa M. Lopes, Michael Pleschka, Alina C. Hilger, Sophia Schneider, William G. Newman, Glenda M. Beaman, Agneta Nordenskjöld, Anne-Karoline Ebert, Martin Promm, Wolfgang H. Rösch, Raimund Stein, Karin Hirsch, Frank-Mattias Schäfer, Eberhard Schmiedeke, Thomas M. Boemers, Martin Lacher, Dietrich Kluth, Jan-Hendrik Gosemann, Magnus Anderberg, Gillian Barker, Gundela Holmdahl, Göran Läckgren, David Keene, Raimondo M. Cervellione, Elisa Giorgio, Massimo Di Grazia, Wouter F. J. Feitz, Carlo L. M. Marcelis, Iris A. L. M. Van Rooij, Arend Bökenkamp, Goedele M. A. Beckers, Catherine E. Keegan, Amit Sharma, Tikam Chand Dakal, Lars Wittler, Phillip Grote, Nadine Zwink, Ekkehart Jenetzky, Alfredo Brusco, Holger Thiele, Michael Ludwig, Ulrich Schweizer, Adrian S. Woolf, Benjamin Odermatt, Heiko Reutter

**Affiliations:** ^1^Institute of Human Genetics, University Hospital Bonn, Bonn, Germany; ^2^Institute for Anatomy and Cell Biology, University Hospital Bonn, University of Bonn, Bonn, Germany; ^3^Department of Pediatrics, Children’s Hospital Medical Center, University Hospital Bonn, Bonn, Germany; ^4^Institut für Biochemie und Molekularbiologie, Universitätsklinikum Bonn, Rheinische Friedrich-Wilhelms-Universität Bonn, Bonn, Germany; ^5^Institute of Neuropathology, University of Bonn Medical Center, Bonn, Germany; ^6^Institute of Pathology, University Hospital Düsseldorf, Düsseldorf, Germany; ^7^Division of Cell Matrix Biology and Regenerative Medicine, Faculty of Biology Medicine and Health, School of Biological Sciences, University of Manchester, Manchester, United Kingdom; ^8^Department of Neonatology and Pediatric Intensive Care, Children’s Hospital Medical Center, University Hospital Bonn, Bonn, Germany; ^9^Centre for Genomic Medicine, Manchester University NHS Foundation Trust, Manchester Academic Health Science Centre, Manchester, United Kingdom; ^10^Department of Women’s and Children’s Health, Center for Molecular Medicine, Karolinska Institute, Stockholm, Sweden; ^11^Pediatric Surgery, Astrid Lindgren Children’s Hospital, Karolinska University Hospital, Stockholm, Sweden; ^12^Department of Urology and Pediatric Urology, University Hospital of Ulm, Ulm, Germany; ^13^Department of Pediatric Urology, Clinic St. Hedwig, University Medical Center Regensburg, Regensburg, Germany; ^14^Medical Faculty Mannheim, Centre for Pediatric, Adolescent and Reconstructive Urology, University Medical Center Mannheim, Heidelberg University, Mannheim, Germany; ^15^Division of Pediatric Urology, Department of Urology, University of Erlangen-Nürnberg, Erlangen, Germany; ^16^Department of Pediatric Surgery and Urology, Cnopfsche Kinderklinik, Nürnberg, Germany; ^17^Department of Pediatric Surgery and Urology, Center for Child and Youth Health, Klinikum Bremen-Mitte, Bremen, Germany; ^18^Department of Pediatric Surgery and Pediatric Urology, Children’s Hospital of Cologne, Cologne, Germany; ^19^Department of Pediatric Surgery, University of Leipzig, Leipzig, Germany; ^20^Department of Pediatric Surgery, Skane University Hospital Lund, Lund, Sweden; ^21^Department of Women’s and Children’s Health, Uppsala Academic Children Hospital, Uppsala, Sweden; ^22^Department of Pediatric Surgery, Queen Silvias Children’s Hospital, Gothenburg, Sweden; ^23^Pediatric Urology, University Children’s Hospital, Uppsala, Sweden; ^24^Pediatric Urology, Royal Manchester Children’s Hospital, Central Manchester University Hospitals NHS Foundation Trust, Manchester, United Kingdom; ^25^Department of Medical Sciences, University of Torino, Turin, Italy; ^26^Medical Genetics Unit, Città della Salute e della Scienza University Hospital, Turin, Italy; ^27^Pediatric Urology Unit, Fondazione Istituto di Ricovero e Cura a Carattere Scientifico Ca’ Granda-Ospedale Maggiore Policlinico, Milan, Italy; ^28^Division of Pediatric Urology, Department of Urology, Radboudumc Amalia Children’s Hospital, Nijmegen, Netherlands; ^29^Department of Genetics, Radboud University Nijmegen Medical Center, Nijmegen, Netherlands; ^30^Department for Health Evidence, Radboud Institute for Health Sciences, Radboud University Medical Center, Nijmegen, Netherlands; ^31^Emma Children’s Hospital, Amsterdam University Medical Center, Vrije Universiteit Amsterdam, Amsterdam, Netherlands; ^32^Department of Urology, Amsterdam University Medical Center, Vrije Universiteit Amsterdam, Amsterdam, Netherlands; ^33^Division of Genetics, Department of Pediatrics, University of Michigan, Ann Arbor, MI, United States; ^34^Department of Human Genetics, University of Michigan, Ann Arbor, MI, United States; ^35^Department of Neurology, University Hospital Bonn, Bonn, Germany; ^36^Department of Ophthalmology, University Hospital Bonn, Bonn, Germany; ^37^Department of Biotechnology, Mohanlal Sukhadia University Udaipur, Udaipur, India; ^38^Department of Developmental Genetics, Max Planck Institute for Molecular Genetics, Berlin, Germany; ^39^Institute of Cardiovascular Regeneration, Center for Molecular Medicine, Goethe University, Frankfurt am Main, Germany; ^40^Department of Pediatric and Adolescent Psychiatry and Psychotherapy, University Medical Centre, Johannes Gutenberg University of Mainz, Mainz, Germany; ^41^Institute of Integrative Medicine, Witten/Herdecke University, Herdecke, Germany; ^42^Cologne Center for Genomics, University of Cologne, Cologne, Germany; ^43^Department of Clinical Chemistry and Clinical Pharmacology, University of Bonn, Bonn, Germany; ^44^Royal Manchester Children’s Hospital, Manchester University NHS Foundation Trust, Manchester Academic Health Science Centre, Manchester, United Kingdom; ^45^Institute for Neuroanatomy, University Hospital Bonn, University of Bonn, Bonn, Germany

**Keywords:** *SLC20A1*, urinary tract development, kidney formation, zebrafish development, cloacal malformation, functional genetics, CAKUT, bladder exstrophy-epispadias complex

## Abstract

Previous studies in developing *Xenopus* and zebrafish reported that the phosphate transporter *slc20a1a* is expressed in pronephric kidneys. The recent identification of *SLC20A1* as a monoallelic candidate gene for cloacal exstrophy further suggests its involvement in the urinary tract and urorectal development. However, little is known of the functional role of *SLC20A1* in urinary tract development. Here, we investigated this using morpholino oligonucleotide knockdown of the zebrafish ortholog *slc20a1a*. This caused kidney cysts and malformations of the cloaca. Moreover, in morphants we demonstrated dysfunctional voiding and hindgut opening defects mimicking imperforate anus in human cloacal exstrophy. Furthermore, we performed immunohistochemistry of an unaffected 6-week-old human embryo and detected *SLC20A1* in the urinary tract and the abdominal midline, structures implicated in the pathogenesis of cloacal exstrophy. Additionally, we resequenced *SLC20A1* in 690 individuals with bladder exstrophy-epispadias complex (BEEC) including 84 individuals with cloacal exstrophy. We identified two additional monoallelic *de novo* variants. One was identified in a case-parent trio with classic bladder exstrophy, and one additional novel *de novo* variant was detected in an affected mother who transmitted this variant to her affected son. To study the potential cellular impact of *SLC20A1* variants, we expressed them in HEK293 cells. Here, phosphate transport was not compromised, suggesting that it is not a disease mechanism. However, there was a tendency for lower levels of cleaved caspase-3, perhaps implicating apoptosis pathways in the disease. Our results suggest *SLC20A1* is involved in urinary tract and urorectal development and implicate *SLC20A1* as a disease-gene for BEEC.

## Introduction

The recent identification of the phosphate transporter *SLC20A1* as a candidate gene for cloacal exstrophy (CE) (OMIM 258040) suggests its involvement also in the lower urinary tract and urorectal development (Reutter et al., 2016). While previous studies in developing *Xenopus* and zebrafish (zf) reported the expression pattern of *slc20a1a* in pronephric kidneys (Nichane et al., 2006; Raciti et al., 2008; Howe et al., 2012; Zhang et al., 2017), the possible role of *SLC20A1* in urinary tract formation is unknown. *SLC20A1* encodes for the sodium-phosphate symporter called the solute carrier family 20 member 1 (PiT-1). The *SLC20A1* protein comprises 12 transmembrane domains (TMDs) (O’Hara et al., 1990; Farrell et al., 2009). *Slc20a1* (*PiT-1*) knock-out mice die by embryonic day 12 (Beck et al., 2009; Festing et al., 2009). While the exact cause of death is unknown, they have gross defects in yolk sac vascular development putatively correlating with *Slc20a1* regulating endocytosis and microautophagy within yolk sac visceral endoderm (Beck et al., 2009; Festing et al., 2009; Wallingford and Giachelli, 2014).

Due to their ease of molecular manipulation and real-time observation, and the high fecundity of the species, we successfully used zebrafish larvae (zfl) to functionally characterize dominant variants reported in individuals with lower urinary tract obstruction (Kolvenbach et al., 2019). Here, we apply a similar zf model to investigate the role of *slc20a1a* in the zf urinary tract and urorectal development, and we combine this with human genomic, cell culture, and immunohistochemistry with regard to *SLC20A1.* Our results suggest *SLC20A1* is involved in human and zf urinary tract and urorectal development, and implicate *SLC20A1* as a disease-gene for bladder exstrophy-epispadias complex (BEEC). Our study also provides early cell culture data to suggest that *SLC20A1* variants found in patients affect cleaved caspase-3, consistent with a reported role of *SLC20A1* in tumor necrosis factor-induced apoptosis (Salaün et al., 2010).

## Materials and Methods

### Zebrafish Husbandry and Embryo Preparation

Zf were kept according to national law and to recommendations by Westerfield (Westerfield, 2000) in our fish facility. Zfl of wild-type AB/TL and transgenic strain *Tg(wt1b:eGFP)* (Perner et al., 2007) were gained by natural fish spawning and raised at 28 C in Danieau (30%) medium on a 14 h light:10 h dark cycle. All zf experiments were performed at ≤ 5 dpf before independent feeding. To suppress pigmentation for later, WISH analysis or fluorescent microscopy 1-phenyl-2-thiourea (final concentration 0.003%) was added to the Danieau solution for respective zfl from 1 dpf onward. Staging of zfl was performed according to Kimmel et al. (1995).

### Microinjections of Morpholino Oligonucleotides and mRNA

Zebrafish embryos were collected 15 min after breeding and were pressure injected into the yolk at the one-cell stage (up to 30–40 min after fertilization) with an ATG-blocking Morpholino^®^ antisense oligonucleotide (MO) by GeneTools, LLC. Injections were carried out with 0.75 ng of *slc20a1a* MO (1.7 nL/embryo) (5′CTGGAGAAAAACACTTCTGGCCTAC 3′) and 0.75 ng of standard control MO (5′CCTCTTACCTCAGTTACAATTTATA 3′). For pressure injection, we used the “Milli-Pulse Pressure Injector, Model MPPI-3” (Applied Scientific Instrumentation, Inc. 29391 W. Enid Rd. Eugene, OR 97402-9533, United States). The MPPI-3 is a self-contained device for producing gas pressure pulses to an injection needle (pulled glass capillary–GB120F-10, Science Products GmbH). The unit offers linear control of both pressure and pulse duration.

### Morpholino (MO) mRNA Rescue

Rescue experiments were performed by co-injection of MO together with 35 pg of human *SLC20A1* polyA mRNA. *In vitro* transcription of *SLC20A1* mRNA was performed using mMessage mMachine Kit (Ambion 1340M) and Poly-A-Tailing-Kit (Ambion AM1350) on IMAGE-clone: 3918690. *slc20a1a* MO sequence is not homologous to the h*SLC20A1* mRNA (0% homology), excluding binding of the MO to h*SLC20A1* mRNA (Supplementary Data Sheet S16).

### Western Blot (WB) Analysis in *slc20a1a* MO Knockdown (KD) zfl

After grading, zfl were pooled into samples of 20–30 larvae of equal grades and lysed in RIPA buffer on ice with 4% protease inhibitor using a sonicator. Proteins were separated by SDS-PAGE, transferred on PVDF membranes and were probed with anti-*SLC20A1* (1:1000; Sigma-Aldrich; AV43905) at 4°C overnight. Enhanced chemiluminescent (ECL) HRP substrate for low-femtogram-level detection was used.

### Sulforhodamine 101 (SR101) Excretion Assay

Excretion assay was performed on days 4–5 dpf. Zfl were kept in the dark in 0.02 mM SR101 in Danieau 30% + 0.003% 1-phenyl-2-thiourea solution for 1 h. After incubation zfl were washed with Danieau 30% three times for 10 min before imaging.

### Whole-Mount Zebrafish *in situ* Hybridization (WISH)

cDNA plasmids for the preparation of antisense and sense probes for *pax2a, evx1, slc20a1a*, and *slc20a1b* were generated by PCR from zebrafish poly-T embryonic cDNA (primer sequences are provided in Supplementary Data Sheet S15). The resulting amplified PCR products were cloned into SK(-) pBluescript^®^. Constructs were linearized by corresponding restriction enzymes and DIG-labeled sense and anti-sense RNA was synthesized using Roche DIG RNA Labeling Kit (Cat. No. 11 175 025 910). WISH was performed following modified instructions of Thisse and Thisse (2008).

### Immunohistochemistry in Whole-Mount Zfl

Zfl were fixed in 4% paraformaldehyde overnight at 4°C and washed afterward with methanol in increasing concentration (25, 50, 75, 100%). Heat-induced antigen retrieval was performed in Tris-HCL (pH = 8.5) at 70°C for 15 min. For permeabilization digest with Proteinase K at room temperature was adapted to age of the zfl. They were then incubated in primary antibodies for 3 days at 4°C (1:500; Anti-Acetulated Tubulin: Sigma-Aldrich – T7451, mouse; Anti-GFP: Invitrogen – A11122, rabbit) and secondary antibodies for 2 days at 4°C (1:1000; Alexa Fluor 546 goat anti-mouse: LifeTechnologies – A11030; Alexa Fluor 488 goat anti-rabbit: LifeTechnologies – A11034).

### High Resolution *in vivo* Fluorescent Zfl Imaging

Embryos were pre analyzed under a Nikon AZ100 Macro-Zoom microscope and selected embryos were further anesthetized with 0.016% tricaine, mounted in 2% low-melting agarose and imaged by two-photon scanning fluorescence *in vivo* microscopy (LaVision Trim-Scope II; ImSpector and ImageJ software).

### Statistical Analysis

Statistical analysis was performed using GraphPad Prism version 8.0.0 for Mac, GraphPad Software, San Diego, CA, United States^1^. Differences with a *p*-value of < 0.05 (^∗^) were considered as being statistically significant. Error bars show standard deviation (SD) in all experiments.

### Human Embryo Immunohistochemistry

Human tissues, collected after maternal consent and ethical approval (REC 08/H0906/21 + 5), were provided by the MRC and Wellcome Trust Human Developmental Biology Resource^2^. Paraffin sections were processed for immunostaining after antigen retrieval, essentially as described (Kolvenbach et al., 2019). Sections were probed with antibody to *SLC20A1* (1:200; Proteintech 12423-1-AP).

### Resequencing of *SLC20A1* in Individuals With BEEC

The resequencing study was conducted in adherence to the Declaration of Helsinki. Informed consents were obtained from affected individuals or by proxies in the case of minors. The study was approved by the ethics committee of the medical faculty of the University of Bonn (No. 031/19) as well as the respective ethic committee of the collaborating centers in Manchester (United Kingdom), Nijmegen (AGORA data- and biobank; Netherlands; Rooij et al., 2016), Torino (Italy), and Stockholm (for Sweden on behalf of Lund, Göteborg, and Uppsala). For resequencing, 690 (440 male and 250 female) BEEC individuals were included [epispadias *n* = 42; classic bladder exstrophy (CBE) *n* = 564; CE *n* = 84]. All three human *SLC20A1* protein coding transcripts (ENST00000272542.7, ENST00000423633.5, ENST00000433924.5) listed in “ensembl database” (September 30, 2017)^3^ were sequenced. PCR-amplified DNA products (primer sequences are provided in Supplementary Data Sheet S15) were subjected to sequencing using a 3130XL Genetic Analyzer (Applied Biosystems, Foster City, United States).

### Generation of Variants for *in vitro* Analysis

QuikChange Lightning Site-Directed Mutagenesis Kit (Agilent #210518) was used to generate variants from IMAGE clone 3918690 (primer sequences are provided in Supplementary Data Sheet S15).

### Cloning for Cell Culture Experiments

N-terminally Flag tagged wild type (wt) and mutant *SLC20A1* cDNA of human origin were cloned into pcDNA3 plasmid backbone (Clontech) (primer sequences are provided in Supplementary Data Sheet S15).

### Cell Culture and Transient Transfection

HEK293 cells were cultured in DMEM/F12 (1:1) (GIBCO) + 10% fetal calf serum (FCS; GIBCO) + 1% penicillin (5,000 U/ml)/streptomycin (5,000 μg/ml). Cells were seeded 1:1 into 24 and six well plates, followed by transient transfection with 250–1,000 ng plasmid DNA per cm^2^ well surface using PANfect A transfection reagent (PAN Biotech, Germany). The experiments were performed 48 h after transfection.

### Western Blot Analysis in HEK293 Cells

Transient transfected HEK293 cells were harvested and lysed in 75 μl homogenization buffer (250 mM sucrose; 20 mM HEPES; 1 mM EDTA in distilled H_2_O, pH 7.4) with 1 mM dithiothreitol; 100 μg of whole cell lysates were separated on 10% sodium dodecyl sulfate (SDS) gels, transferred on nitrocellulose membranes, and probed with antibodies against FLAG-tag (1:1,000; Sigma Aldrich; F3165) and ß-ACTIN (1:40,000; Sigma Aldrich; A3854). Non-transfected HEK293 cells served as negative control. For analysis of cleaved caspase-3 (CC3) levels 20 μg of cell lysates were loaded and probed with anti-CC3 (1:2,000; Cell Signaling; 9661) and ß-ACTIN (1:10,000; Sigma Aldrich; A5441). Analysis of proliferation cell nuclear antigen (PCNA) levels was performed by blotting 2 μg of cell lysates of all respective groups and probing those with anti-PCNA (1:5,000; Abcam; ab2426) and ß-ACTIN. ImageJ was used for WB densitometry.

### Phosphate Uptake Assay in Human Embryonic Kidney 293 (HEK293) Cells

Transient transfected HEK293 cells seeded on 24 well plates were incubated in uptake buffer (96 mM NaCl; 2 mM KCl; 1.8 mM CaCl_2_; 1 mM MgCl_2_; 50 mM HEPES in distilled H_2_O) supplemented with 200 μM potassium phosphate buffer and 1 μCi ^32^PO_4_^3–^ per ml uptake buffer for 3and 15 min before washing, respectively. Cell-associated radioactivity was measured with a ß-counter (Tri-Carb^®^ Liquid Scintillation Analyzer, Perkin Elmer). Values are given as counts per minute and calculated as percentage. Non-transfected HEK293 cells served as background control.

### Computational 3D Structural Modeling

For 3D structure, modeling of human *SLC20A1* variants (Uniprot ID: Q8WUM9) I-Tasser^4^ was used. This employs an integrated combinatorial approach comprising comparative modeling, threading, and *ab initio* modeling (Roy et al., 2010) using the procedure adopted by Dakal et al. (2017). The generated structures were visualized in Chimera 1.13rc version.

## Results

### Morpholino KD of *slc20a1a* in Embryonic Zebrafish

**FIGURE 1:**
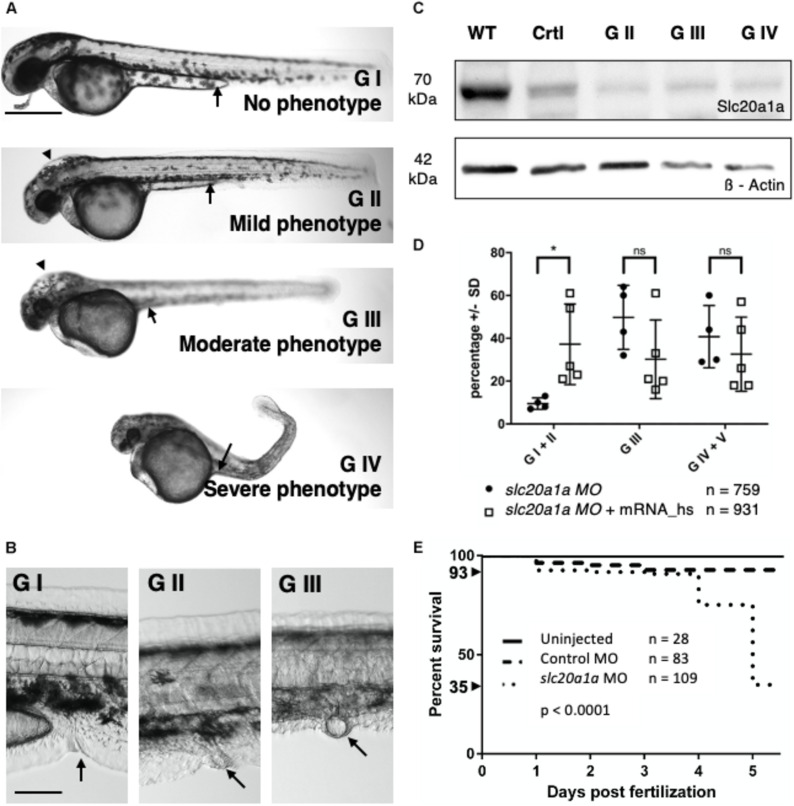
*slc20a1a* MO KD. KD was performed by injecting 0.75 ng of ATG-blocking MO into one-cell-staged wt zf eggs. Lateral view, dorsal to top, cranial on the left. **(A)** Phenotypical grading of MO injected zfl at 2 dpf in four grades increasing in severity and lethality. Scale bar: 500 μm. See main Results text for grading details. In brief, GI were normal in direct inspection; GII had mild defects; GIII had moderate defects, and GIV had severe defects. G V resembles dead zfl. Supplementary Data Sheet S3 shows close-ups for better demonstration of hydrocephalus and eye development in all four grades presented. **(B)** Examples for cloacal abnormalities in MO KD zfl at 2 dpf. Scale bar: 100 μm. Inspection of cloacal region after previous grading revealed malformations in cloacal region in G II and G III sorted zfl increasing in amount and severity with grading. G I sorted zfl had normal cloacal morphology, that is, a thin and curved organ with a distal opening (arrow). GII and GIII zfl have abnormally shaped cloacae, with dilated and/or apparently blind-ending lumens. Further cloacal close-ups of G II and III sorted zfl are shown in Supplementary Data Sheet S4. **(C)** WB shows efficacy of MO KD in zebrafish protein lysates from 2 dpf. 70 kDa: *slc20a1a*, 42 kDa: ß-Actin loading control; WT = uninjected control, Crtl = control MO injected, G II/III/IV = *slc20a1a* MO KD zfl sorted by grading II–IV. *Slc20a1a* can be detected in uninjected wt control and injected control MO group. Only a weak *slc20a1a* signal was seen in MO KD groups G II, III, and IV, which showed phenotypical features as described in **(A)**. WB shows correlation between phenotype and protein expression. Raw data of WB is shown in Supplementary Data Sheet S6. **(D)** Co-injection of *slc20a1a* MO with 35 μg *in vitro* transcribed human *SLC20A1* polyA mRNA shows partial rescue of various phenotypes underlining the Morpholino’s specificity. *n* = 5 (here *n* = 1 represents the average score in each experimental batch). Error bars show SD. *X*-axis shows groups at 3 dpf that were compared: zfl showing no or only a very mild phenotype (G I + G II), larvae with a moderate phenotype (G III) and a last group of larvae with a severe and lethal phenotype (G IV) together with those who were already dead at time of comparison (G V). *Y*-axis shows the percentage of zfl in the corresponding groups described before. A significant difference (^∗^*p* = 0.01) was seen within the first group of MO and MO-mRNA rescue group, showing a partial rescue of *slc20a1a* MO KD phenotype reflected in bigger group of phenotypically not or only mildly affected zfl. Mere overexpression of *SLC20A1* wt mRNA in zfl resulted in phenotypical aberrations, which did not fit the grading characteristics and the phenotypes observed in *slc20a1a* MO KD. Further, pure *SLC20A1* overexpression in zfl resulted in higher lethality compared to non-injected control groups (data not shown). These findings suggest that the MO rescue effect of *SLC20A1* wt mRNA is weakened and disguised by the mRNA’s general negative overexpression effect. **(E)** Kaplan-Meyer curve shows significantly reduced survival (*p* < 0.0001) in *slc20a1a* MO-injected group compared to uninjected and control MO. Survival rates by day five post-fertilization: WT = 100%, control MO injected group = 93%, MO injected group = 35% (including all grades). Exclusion of embryos dying/not developing by 8 h post-fertilization (hpf) due to failed fertilization or consequences of tissue damage caused by injections with mechanical manipulation. Within these initial 8 hpf intervals, no difference was seen between control MO and *slc20a1a* MO injected groups.

To further functionally characterize *SLC20A1* we performed ATG-blocking MO KD experiments in zfl. The zf has two ortholog genes, *slc20a1a* and *slc20a1b*. Focusing on the segment of chromosome 2 harboring the *SLC20A1* locus in humans, none of the distinct chromosome loci of the two zf orthologs show conserved syntenies to the human segment. NCBI Unigene’s EST profile viewer reveals distinct expression patterns for both zf orthologs (Nichane et al., 2006): only *slc20a1a* appears to be strongly and specifically expressed in the embryonic kidney. While in the developing zfl no specific expression has been described for *slc20a1b* so far (January 2020)^5^, clearly *slc20a1a* has been established as a pronephric tubular marker (Howe et al., 2012; Zhang et al., 2017). Here, WISH analysis confirmed *slc20a1a* as a pronephric marker, with expression in the proximal (i.e., near to the glomerulus) section, while WISH analysis of *slc20a1b* did not show any signal in tissues relevant to urinary tract development (Supplementary Data Sheets S1A–F). Hence, we studied MO KD against *slc20a1a* to characterize its possible developmental impact on urinary tract and urorectal development. The injection procedure was uniform in all experiments. *slc20a1a* MO injected larvae showed a range of phenotypes on direct inspection. To facilitate analyses, we graded all MO injected zfl at 2 dpf (Long-pec, according to Kimmel et al., 1995) on the basis of several phenotypical features. Representative images of the grades (G) are depicted in Figure 1. In brief: G I embryos (5% at 2 dpf) appeared normal and identical to uninjected and control MO injected zfl; G II zfl (6% at 2 dpf) showed a mild phenotype with minimal reduction in body and head sizes, no or moderate hydrocephalus, mild pigmentations defects, and benign reduction of the yolk sac extension, but without changes in body curvature or eye abnormalities; G III zfl (46% at 2 dpf) showed a moderate phenotype characterized by decreases in body and head sizes, overt hydrocephalus (arrow heads, additional images in Supplementary Data Sheet S3), yolk endocytosis defects, lack of yolk sac extension along abdominal wall (arrows), eye abnormalities especially in size (Supplementary Data Sheet S3), straight body with or without kinking of the tail tip (no kinking shown here), pigmentation defects, and pericardial effusion; and G IV zfl (27% at 2 dpf) had marked malformations of various organ systems and additional defects in body curvature. Remarkably, no G IV larvae survived until 5 dpf and were therefore not further analyzed but scored as “dead” (G V) in the statistics presented. Supplementary Data Sheet S3 shows close-ups for better demonstration of hydrocephalus and eye development in all four grades presented.

### Cloacal Anomalies in *slc20a1a* MO KD

Though cloacal anomalies were not part of the preceding grading, we frequently found malformations in the cloacal region and therefore the urinary outflow tract in mild (GII) and moderate (GIII) *slc20a1a* MO KD zfl (Figure 1B). These cloacal malformations could themselves be graded as moderate or severe (additional images in Supplementary Data Sheet S4). G I sorted zfl had normal cloacal morphology, that is, a thin and curved organ with a distal opening (arrow). G II and G III zfl has abnormally shaped cloacae, with dilated and/or apparently blind-ending lumens. Further cloacal close-ups of G II and III sorted zfl are shown in Supplementary Data Sheet S4. Importantly, the finding of cloacal anomalies in larvae that had only a mild whole-body phenotype suggests that the former is a “strong” primary effect and not simply a side effect of a more major whole-body malformation. For further characterization, we performed WISH in *slc20a1a* MO, control MO, and uninjected wt zfl with two different cloacal marker probes at two timepoints each. *pax2a* marks the distal part of the pronephric ducts up to their fusion at the cloaca. For quantification of *pax2a* expression, we determined the maximal distance orthogonal to the pronephric midline within the stained cloacal region (Supplementary Data Sheets S5A–C). *evx1* is a WISH marker for the cloaca. The area of staining was measured using a common threshold in all zfl (Supplementary Data Sheets S5D–F). For *pax2a*, a wider cloaca was found in *slc20a1a* MO KD zfl at both timepoints. Cloacal area of expression of *evx1* was significantly larger in *slc20a1a* MO KD zfl at both timepoints as well. These results suggest a defect in tissue development in the cloacal region of *slc20a1a* MO KD zfl.

### Efficiency and Specificity of MO KD Shown by WB Analysis and mRNA Rescue

Efficacy of *slc20a1a* MO KD, at the protein level, was demonstrated by WB analysis at 2 dpf (Figure 1C). *Slc20a1a* protein was detected in both control groups at about 70 kDa molecular weight. We could only detect weak *slc20a1a* protein signal in the MO KD grades (G II–IV).

To test the specificity of our *slc20a1a* MO, we co-injected *in vitro* transcribed polyA mRNA of human wt *SLC20A1*. We detected a rescue effect of the human *SLC20A1* mRNA in MO zfl, as evidenced by significant increase of the proportion of overtly normal or mildly affected zfl (GI + GII) (Figure 1D). This effect was even more notable given the fact that overexpression of *SLC20A1* wt mRNA in non-morphant zfl resulted in higher lethality and phenotypical aberrations, which did not fit the grading characteristics and the phenotypes observed in *slc20a1a* MO KD. Collectively, the results support the *slc20a1a* MO’s specificity.

Survival of uninjected wt, control MO, and *slc20a1a* MO KD zfl were monitored until 5 dpf, showing a significant decrease (>50%) in survival rate in the MO KD larvae (Figure 1E).

### Pronephric Cysts and Dilatation of Pronephric Ducts in *slc20a1a* MO KD Implicate *slc20a1a* as Important Player in Early Kidney Development

**FIGURE 2:**
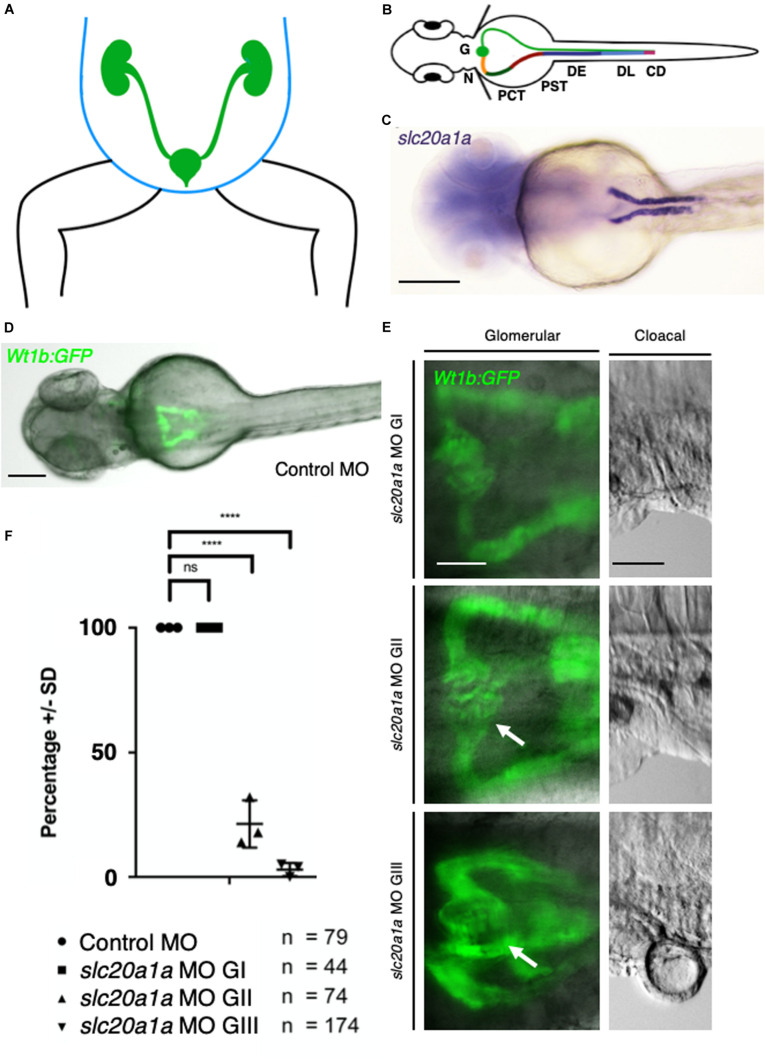
Pronephric cysts in *slc20a1a* MO KD resulting from cloacal obstruction due to malformations. **(A)** Scheme of human abdomen, green: urogenital tract with kidneys, ureter, bladder, urethra; blue: abdominal wall. **(B)** Pronephros in zfl (green: right side) as equivalent to human urinary tract. Scheme of zfl, dorsal view at (2 dpf, patterning of zebrafish pronephros is similar to human nephron segmentation. Specific segments are color coded (left side) for better identification: G, Glomerulus; N, Neck; PCT, Proximal Convoluted Tubule; PST, Proximal Straight Tubule; DE, Distal Early; DL, Distal Late; CD, Collecting Duct. **(C)** Whole mount *in situ* hybridization (WISH) against *slc20a1a* in zfl at 2 dpf, labeling proximal part of pronephros. Scale bar: 500 μm. **(D)** Control MO zfl in *Tg(wt1b:GFP)* (Perner et al., 2007) marking proximal part of pronephros, Scale bar: 100 μm. **(E)** Glomerular close-ups in *Tg(wt1b:GFP)* zfl in dorsal view at 2 dpf (Scale bar: 50 μm). On the left, pronephric cysts (arrows) and dilatation of proximal part of pronephros increasing in severity with grading are shown. G I (upper pictures) showing no cystic phenotype, G II (middle pictures) showing a mild cyst formation, and G III (bottom pictures) showing severe cysts and a wide dilatation of the pronephros. Corresponding cloacal close-ups, shown on the right in lateral view (Scale bar: 50 μm), underline correlation between malformations in urinary outflow tract and cysts as well as pronephric dilatations in corresponding groups. **(F)** Graph shows percentage of zfl (*Y*-axis) in the following groups: Control MO, *slc20a1a* MO G I, *slc20a1a* MO G II, *slc20a1a* MO G III (*X*-axis). Each dot stands for one individual experiment (here *N* = 1 represents the average score in each experimental batch). Whereas control MO and *slc20a1a* MO G I larvae do not show any cystic phenotype, only an average of 21% of MO G II, and solely 3% of G III MO zfl show normal configuration of the proximal part of pronephros. The graph shows significant differences between control MO and phenotypically normal group MO G I compared to MO G II and G III, *N* = 3, ****p < 0.0001. Error bars show SD.)

In zfl, the pronephros represents the anatomical structure that corresponds to the human urinary tract. The pronephric pattern is analogous to the mammalian nephron. The two pronephric ducts fuse and excrete the urine through the cloacal opening (Figures 2A,B). Figure 2C shows clear WISH signal for *slc20a1a* in the proximal zfl pronephros at 2 dpf. *slc20a1a* WISH in earlier developmental zfl stages show expression in intermediate mesoderm, which later forms the pronephros, red blood cells, and trunk endothelium (Supplementary Data Sheet S1). We used *Tg(wt1b:eGFP)* reporter fish (Perner et al., 2007) to assess the impact of *slc20a1a* on the development of the glomeruli and the proximal region of the pronephros (Figure 2D). Whereas control MO and *slc20a1a* MO G I zfl did not show any phenotypical differences concerning the morphology of the glomeruli and pronephros at 2 dpf, the majority of G II (79%) and G III (97%) sorted larvae showed glomerular cysts and a dilatation of the proximal part of the pronephric ducts (Figures 2E,F).

We sought cilia within the pronephros using immunofluorescence staining against alpha-acetylated tubulin and GFP in Tg(wt1b:GFP) at 2 dpf. Our two-photon microscopy (Supplementary Data Sheet S7) revealed the presence of cilia. There were no gross structural anomalies, such as gross shortening or elongation, between the G II and G III MO-KD zfl. We did not, however, formally quantify cilia length nor did not assess motility study in G II and G III MO-KD zfl. The dilatation of their pronephric ducts was confirmed.

Dilatation of the ureter and pelvis of the kidney in human can be caused by a backlog of urine due to a functional or anatomical blockage of the urinary tract. This blockage occurs usually distal to the dilated parts of the renal tract. *slc20a1a* MO KD zfl show malformations in the urinary outflow tract (Figures 1B, 2E and Supplementary Data Sheet S4). Here, malformations of urinary outflow tract in morphants correlate with severe cystic dilatation of the pronephric kidney. This strongly supports the hypothesis that the cloacal malformations seen in *slc20a1a* MO KD zfl cause a backlog of urine, which leads to cystic and dilated pronephric kidneys.

### Sulforhodamine 101 Excretion Assay in *slc20a1a* MO KD zfl Shows Gut Outlet Obstruction at the Cloaca

**FIGURE 3:**
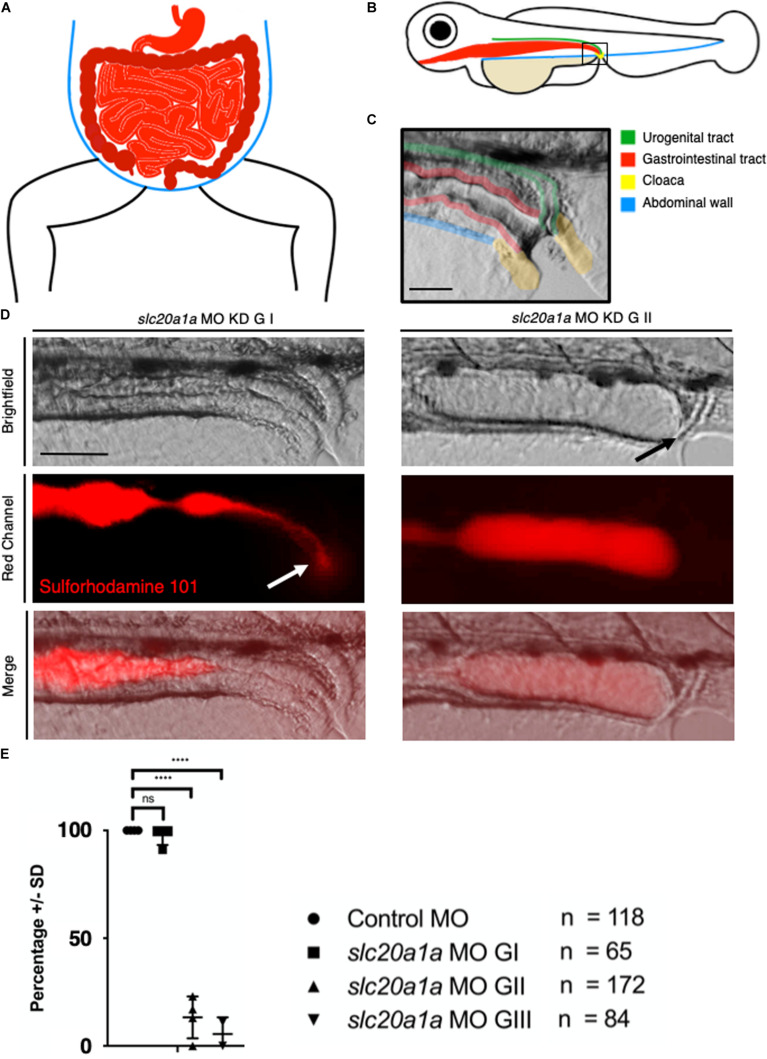
Sulforhodamine 101 excretion assay shows imperforate hindgut in *slc20a1a* MO KD zfl. **(A)** Scheme of human abdomen, red = GIT with stomach, duodenum, jejunum, ileum in light red and colon, rectum, anus in dark red; blue = abdominal wall. **(B)** Scheme of zfl, lateral view at 5 dpf; green = urogenital tract ((pronephros), red = GIT, blue = abdominal wall, beige = yolk sac, yellow = cloaca. **(C)** Cloaca in zfl at 5 dpf: fusion and opening of pronephros and GIT at cloaca between 4 and 5 dpf. Pseudocolored for identification as above in B: green = urogenital tract (pronephros), red = GIT, yellow = cloaca. Scale bar: 50 μm. **(D)** Opening of cloaca and excretion of SR101, a red fluorescent dye labeling the content of zfl intestine. Upper panel shows brightfield, middle panel red channel, lower panel shows a merged view of both channels. On the left we show *slc20a1a* MO KD G I zfl at 5 dpf compared to *slc20a1a* MO KD G II zfl at 5 dpf on the right side of the panel. In control MO and *slc20a1a* MO GI, zfl dye uptake is not disturbed; we could detect clear and bright red dye fluorescence in the gut of all animals. Dye excretion and opening of the cloaca was not disturbed. White arrow marks dye excretion from the cloaca. In contrary, we observed cloacal opening and excretion defects in *slc20a1a* MO KD G II zfl at 5 dpf mimicking an imperforate anus as shown on the right side of the panel. Black arrow marks opening defect and therefore resulting dilatation of intestine due to bag log. No changes in peristalsis of the GIT was observed; hence, expansion of distal part of intestine as shown here is solely caused by lack of cloacal opening. Scale bar: 50 μm. **(E)** Significant differences in opening of cloaca at 5 dpf in zfl between phenotypically affected and control MO. Cloacal opening was monitored for several minutes up to 1 h. Only 13.25% of G II and 5.5% of G III zfl showed cloacal opening and therefore excretion of SR101 from the GIT, whereas 82.5% of G II and 74% of G III zfl did not show any excretion. In the remaining 4.25% of G II and 20.5% of G III, zfl cloacal opening could not be assessed resulting from failure of SR101 uptake in the first place or misshape and tissue malformations not allowing to assess the cloacal region in the respective zfl. *N* = 4, ****p < 0.0001. Error bars show SD.)

Figure 3 depicts the gastrointestinal tract (GIT) and cloacal opening of the hindgut. Schematic comparison of GIT of humans and zf is shown in Figures 3A,B (red). The close-up of the cloacal region in zfl shows opening of pronephros and GIT at cloaca in a healthy zfl at 5 dpf (Figure 3C).

Fluorescent dye (Sulforhodamine 101, SR101) uptake and excretion assay in zfl at 5 dpf showed normal excretion and opening of the cloaca in all control MO zfl and in an average of 98% in *slc20a1a* MO G I zfl (Figures 3D,E). On the contrary, excretion of SR101 was severely disturbed and absent in G II (82%) and G III (74%) *slc20a1a* MO zfl. This assay confirmed the high abundance of cloacal opening defects in *slc20a1a* morphants resembling an imperforate anus in humans. For clarification of the performed dye assay, representative fluorescent videos are provided in the supporting information (Supplementary Data Sheets S8, S9).

### Embryonic Protein Expression of SLC20A1 in Human Embryonic Urogenital Tissue

**FIGURE 4:**
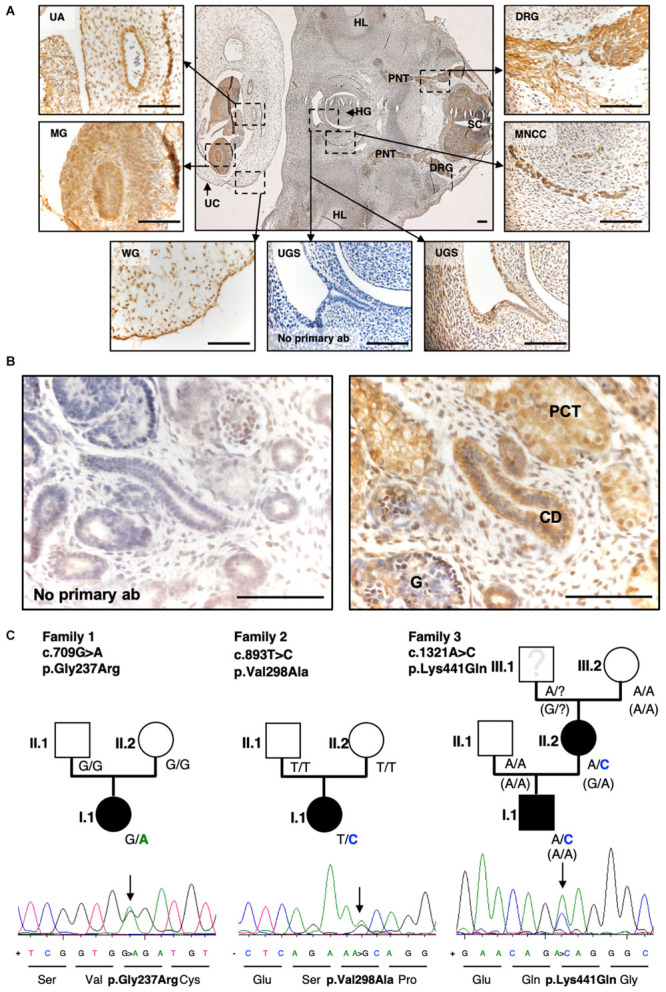
Exome sequencing and targeted Resequencing in families with BEEC phenotype identified disease variants in *SLC20A1.* **(A)** IHC of a transverse section of a 6-week-old human embryo. *SLC20A1* was immunodetected (brown) in the urogenital sinus (UGS), which develops into the urinary bladder. Furthermore, (textitSLC20A1 was also detected in the spinal cord (SC), dorsal root ganglia (DRG), peripheric nerve trunk (PNT), hind limb (HL), hindgut (HG), migrating neural crest cells (MNCC), umbilical cord (UC), Wharton’s jelly (WG), midgut (MG), and umbilical artery (UA). Scale bars = 100 μm. **(B)** Histology section of the human 10-week-gestation metanephric metanephros. Note prominent *SLC20A1* immunostaining (brown) in the proximal tubule (PT) and the collecting duct (CD). The glomerulus (G) shows a fainter signal. Scale bars: 100 μm. **(C)** Exome sequencing of eight CE case-parent-trios revealed *de novo* variant c.709G > A (p.Gly237Arg) in *SLC20A1* in family 1 (Reutter et al., 2016). Resequencing of 690 individuals with BEEC led to identification of two more variants in individuals with CBE: c.893T > C (p.Val298Ala) in family 2 as *de novo* change c.1321A > C (p.Lys441Gln) with maternal inheritance (maternal phenotype: fusion defect of pelvic bone, mild phenotype) in family 3. Pedigrees of all three families are shown with genotypes of all individuals indicated. In family 3, the maternal grandfather (Figure 1B, family 3, person III.1) was not available for testing. For haplotype analysis of all available family members (Figure 1B, family 3, III.2, II.1, II.2, I.1), we used the synonymous marker rs4849091 at chromosomal position chr2:113404708 A > G (p.Leu101=) of the canonical transcript ENST00000272542.7. Genotypes of rs4849091 are shown in brackets. All variants are heterozygote changes and result in missense variants as shown in Sanger sequences including amino acid sequences below each pedigree.)

Figure 4A shows a transverse section of a healthy (non-BEEC) 6-week gestation human embryo. *SLC20A1* was immunodetected in several locations including the urogenital sinus and the urinary bladder precursor implicated in BEEC. Additionally, we immunodetected *SLC20A1* in a 10-week-gestation metanephric kidney, with prominent signals in the proximal tubules and collecting ducts (Figure 4B).

### Resequencing of *SLC20A1* Identifies Two Additional Variants

Resequencing of all three genomic *SLC20A1* transcripts identified an additional *de novo* variant in a case-parent trio (c.893T > C, p.Val298Ala, ENST00000272542.7, allele frequency 0.000003979, Figure 4C, family 2, person I.1) as well as a novel *de novo* variant in an affected mother (Figure 4C, family 3, person II.2) who transmitted this variant to her affected son (c.1321A > C, p.Lys441Gln, ENST00000272542.7, Figure 4C, family 3, person I.1) (Supplementary Data Sheet S10 Table for additional information). In this family, the maternal grandfather of the index person (Figure 4C, family 3, person III.1) was not available for testing. Haplotype analysis of all available family members (Figure 1B, family 3, III.2, II.1, II.2, I.1) showed that the disease variant (c.1321A > C) must have occurred *de novo* in the grandmother’s derived germ cell (Figure 4C, family 3, person III.2). For haplotype analysis, we used the synonymous marker rs4849091 at chromosomal position chr2:113404708 A > G (p.Leu101) of the canonical transcript ENST00000272542.7. According to gnomAD this marker has a MAF of 0.4871 across all ethnicities and resides in exon 8 of *SLC20A1* in proximity of 12 kb to variant c.1321A > C.

### Prediction of Variant Localizations in SLC20A1

**FIGURE 5:**
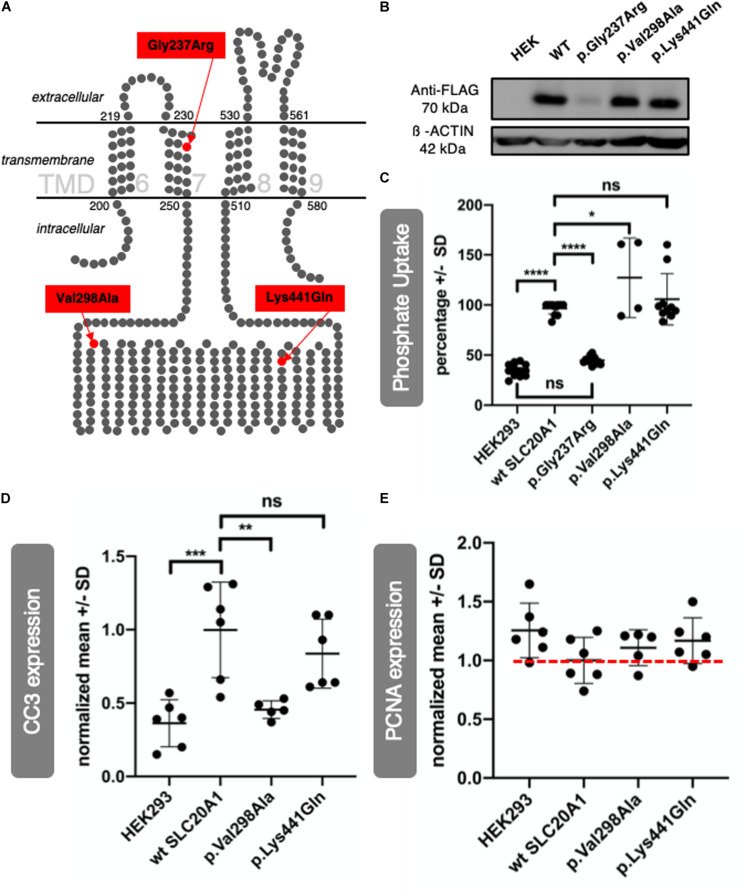
SLC20A1 as transmembrane phosphate transporter and *in vitro* characterization of its variants found in BEEC individuals. **(A)** *In silico* 2D model of *SLC20A1*, a multi-pass integral membrane protein, indicating localization of variants found in affected individuals, c.709G > A (p.Gly237Arg) in TMD 7, c.893T > C (p.Val298Ala) and c.1321A > C (p.Lys441Gln) are located in an intracellular loop. Only four (6–9) of a total of twelve TMDs are shown in this simplified model. The model was generated based on the data of Beck et al. (2009). **(B,C)** *SLC20A1* mediated ^32^PO_4_ transport in transiently transfected HEK293 cells. **(B)** WB analysis of 100 μg whole cell homogenates obtained from HEK293 cells transfected with 500 ng plasmid DNA per cm^2^ well surface. Plasmid DNA was FLAG tagged and transfection efficiency was detected using anti-FLAG antibody (70 kDa) and anti-ß-ACTIN antibody (42 kDa), which served as loading control. As expected, no FLAG signal could be detected in negative control (untransfected HEK293 cells, marked as HEK). Transfection worked for wt *SLC20A1* overexpression as well as the variants c.893T > C (p.Val298Ala) and c.1321A > C (p.Lys441Gln). No FLAG signal could be detected for c.709G > A (p.Gly237Arg) transfected cells. Even when transfected with higher plasmid concentrations, p.Gly237Arg was not detectable in HEK293 cells (Supplementary Data Sheet S12). **(C)** Endpoint assay of transient transfected HEK293 cells. Cells were incubated with 1 μCi ^32^PO_4_^3–^ and 200 μM K_3_PO_4_ for 15 min. For better comparison, a highest number of counts per minute in wt *SLC20A1* overexpression group was set as 100% in each experiment (*N* = 6 with two datasets each), values of HEK293 and variants were calculated correspondingly, and resulting values in percentage are shown on the *y*-axis (Error bars show SD). A two-way ANOVA of the grouped analysis was significant ((*p* < 0.0001). p.Gly237Arg did not show any differences of phosphate uptake to negative HEK293 control. This is in line with the expression deficiency of p.Gly237Arg described before. Wt *SLC20A1* overexpression showed a significant increase of phosphate uptake compared to untransfected HEK293 (Tukey’s multiple comparison: ****p < 0.0001). Amin acid change p.Lys441Gln and p.Val298Ala overexpression resulted in an even higher phosphate uptake than wt *SLC20A1* overexpression with a significant difference between p.Val298Ala and wt *SLC20A1* (Tukey’s multiple comparison: *p < 0.05). Therefore, variant overexpression does not impair phosphate uptake capability *in vitro*. **(D,E)** Densitometric analysis of WBs (*N* = 6) from whole cell homogenates was obtained from transfected HEK293 cells. WBs are provided in Supplementary Data Sheet S13. *Y*-axis shows normalized values against wt *SLC20A1* overexpression. Error bars show SD. **(D)** Expression of CC3 as apoptosis marker was measured in six WBs of corresponding independent transfection experiments. The one-way ANOVA was significant (*p* = 0.0002), Tukey’s multiple comparison test was significant for HEK293 vs. WT (***p = 0.0005), and WT vs. p.Val298Ala (**p = 0.0038). Wt *SLC20A1* overexpression in HEK293 cells increased apoptosis when compared to untransfected negative control (HEK). There is no induction of apoptosis inc.893T > C (p.Val298Ala) transfected cells, comparable to untransfected negative control (HEK). c.1321A > C (p.Lys441Gln) does not result in significant reduction of CC3 expression. However, a trend of reduced CC3 expression in comparison to wt *SLC20A1* overexpression can be seen. **(E)** Same analysis was used to study expression of PCNA as a proliferation marker. A one-way ANOVA did not show significant results (*p* = 0.1903). Nevertheless, wt *SLC20A1* overexpression seems to reduce PCNA expression when compared to negative control (untransfected HEK). Variants analyzed [c.893T > C (p.Val298Ala) and c.1321A > C (p.Lys441Gln)] tend to reduce PCNA less than WT overexpression (red dotted line for better comparison).)

The *SLC20A1* (PiT-1) protein is a sodium-dependent inorganic phosphate (Pi) symporter that contains 12 TMDs (O’Hara et al., 1990; Farrell et al., 2009). Three-dimensional crystal structures of most SLC family transmembrane proteins are unknown (Dakal et al., 2017). Figure 5A shows TMDs 6 to 9 of a putative 2D structure of the *SLC20A1* protein, modeled by Beck et al. (2009). Here, variant c.709G > A (p.Gly237Arg) of family 1 is located in TMD 7, and both variants, c.893T > C (p.Val298Ala), of family 2 and variant c.1321A > C (p.Lys441Gln) of family 3 are located in a large intracellular loop between TMDs 7 and 8. A further attempt of ours to predict localization of the variants in a 3D model of *SLC20A1* protein is shown in Supplementary Data Sheet S11. Our 3D model confirmed the localization of the two variants c.893T > C (p.Val298Ala) and c.1321A > C (p.Lys441Gln) in a large intracellular loop between TMD 7 and 8. The focus is on c.709G > A (p.Gly237Arg), though we predict a possible shift from TMD 7 (as described by Beck et al., 2009) to TMD 6 in our 3D model. In transmembrane proteins, glycine resides in helices, predominantly at the helix-helix interface, which makes it an important structural player (Li and Deber, 1992; Javadpour et al., 1999). In our proposed 3D model, p.Gly237Arg is present at the interface of TMD 6 and TMD 1. Accordingly, p.Gly237Arg might cause instability in TMD 6.

### Functional Characterization of *SLC20A1* Variants *in vitro*

*In vitro* characterization of all three *SLC20A1* variants was performed using HEK293 cells transfected with either human wt *SLC20A1* or one of the three respective variants – all FLAG-tagged. Transfection efficiency was confirmed by WB (Figure 5B). Variant c.709G > A (p.Gly237Arg) was not expressed efficiently in HEK293 cells as protein. A dosage effect was excluded (Supplementary Data Sheet S12). Expression of c.893T > C (p.Val298Ala) and c.1321A > C (p.Lys441Gln) variants resulted in protein levels similar to that of expressed wt *SLC20A1* protein. Phosphate uptake in the transfected cells was measured in a radioactive labeled phosphate assay (Figure 5C). Untransfected HEK293 cells and c.709G > A (p.Gly237Arg) transfected cells displayed a basal phosphate uptake. Cells transfected with either human *SLC20A1* wt, c.893T > C (p.Val298Ala) or c.1321A > C (p.Lys441Gln) had a threefold increased capability of phosphate uptake compared to untransfected control cells. We therefore conclude that *SLC20A1*-linked phosphate uptake is not disturbed by the two *de novo* variants.

Given that *SLC20A1* protein is known to play a role in apoptosis pathways (Salaün et al., 2010; Husseini et al., 2013) we used WB to assess the apoptosis marker CC3 in the above described transfected HEK293 cells. Overexpression of wt *SLC20A1* increased the level of CC3. In order to quantify CC3 detection in WB, we performed densitometry (Figure 5D and Supplementary Data Sheet S13). In contrast to wt *SLC20A1*, variant c.893T > C (p.Val298Ala) failed to increase CC3 levels above those in control cells. Variant c.1321A > C (p.Lys441Gln) increased CC3 levels but to a lesser extent compared to wt *SLC20A1* overexpression. Variant c.709G > A (p.Gly237Arg) was not studied further due to its expression deficiency in HEK cells previously mentioned (Figure 5B and Supplementary Data Sheet S12). We conclude that overexpression of BEEC variants shows differences compared to wt overexpression when analyzing expression of CC3 as an apoptosis marker. Additionally, we performed WB analysis of the proliferation marker PCNA (Figure 5E and Supplementary Data Sheet S13) and found no significant differences in PCNA levels between the experimental groups; yet, our data shows a trend: while wt *SLC20A1* overexpression was associated with a reduced PCNA level, neither c.893T > C (p.Val298Ala) nor c.1321A > C (p.Lys441Gln) had a similar strong effect.

## Discussion

The results of our study suggest that *SLC20A1* is not only involved in embryonic kidney formation but also in urinary tract and urorectal development. Furthermore, our findings suggest that monoallelic *de novo* variants in *SLC20A1* are involved in BEEC formation. These conclusions are supported by the immunodetection of *SLC20A1* in the BEEC relevant developmental organ field comprising the urogenital sinus and early human embryonic kidney. In this context, CBE and CE individuals present with an increased incidence of kidney and upper urinary tract anomalies comprising ureteropelvic junction obstruction, ectopic pelvic kidney, horseshoe kidney, kidney hypo- or agenesis, megaureter, ureteral ectopy, and ureterocele (Stec et al., 2012). Accordingly, we observed dilatation of the proximal part of pronephric ducts in *slc20a1a* MO KD zfl. From our morphological description of *slc20a1a* MO KD, it appears that the observed cystic dilatations and the dilatation of the pronephros are due to urinary backlog caused by pronephric outlet obstruction (Figures 1B, 2E and Supplementary Data Sheet S4). The latter interpretation resembles human hydronephrosis due to vesicoureteral reflux rather than a primary architectural defect of the pronephric mesenchyme.

In accordance with previous work, our WISH analysis confirms *slc20a1a* in zfl as specific pronephric marker in 48 hpf zfl (Figure 2C). Interestingly, expression of *slc20a1a* in zfl in earlier stages is not restricted to the proximal pronephric area but can be seen in the intermediate mesoderm as well as in the cloacal region (Supplementary Data Sheet S1). Whether *slc20a1a* is expressed in the cloacal tissue cannot be confirmed be our current data. Our data suggests *slc20a1a* as regulator of early urinary tract and kidney formation. The wide range of phenotypes that affect several organ systems seen in *slc20a1a* MO KD zfl stresses the importance of *slc20a1a* in early zf development. It not only alters the cloacal formation but leads to developmental defects of the eye, spine, and brain. Interestingly, the description of the phenotypical features of *slc20a1a* MO KD zfl matches phenotypes seen by Nathaniel Abraham (Abraham, 2004). In their study, Abraham used the autophagy specific inhibitor 3 methyladenine (3MA) and caspase 3 inhibitor Z-DEVD-FMK in developing zf embryos to study inhibition of two apoptosis pathways. The striking similarities between those zfl treated by Abraham with 3MA and Z-DEVD-FMK and our *slc20a1a* MO KD zfl suggest a defect in apoptosis due to KD of *slc20a1a*. This is in line with our overexpression studies of identified BEEC variants in HEK293 cells. Overexpression of both newly identified *de novo* variants in HEK293 cells resulted in lower CC3 levels compared to overexpression of wt h*SLC20A1* (Figure 5D and Supplementary Data Sheet S13) suggesting that both variants interfere with apoptosis.

Previous exome sequencing in case-parent-trios with CE identified a novel *de novo* variant in *SLC20A1* (Reutter et al., 2016). Here, we identified two additional *de novo* variants in *SLC20A1* in two independent BEEC families. Of the three *de novo* variants identified so far, the variant with the highest predicted functional impact (c.709G > A, p.Gly237Arg, family 1) locates in TMD 7 of *SLC20A1* (Figures 5A–D model). In the case of transmembrane proteins, glycine resides in helices predominantly at the helix-helix interface and thus plays a major structural role (Li and Deber, 1992; Javadpour et al., 1999). The amino acid change p.Gly237Arg is located in a transmembrane helix, but whether it is lying at a helix-helix interface cannot be determined using the 2D model by Beck et al. (2009). However, in our 3D model, p.Gly237Arg lies in TMD 6 and is present at the interface of TMD 6 and TMD 1 (Supplementary Data Sheet S11), suggesting that this variant might lead to instability in TMD 6. Expression deficiency of c.709G > A (p.Gly237Arg) in HEK293 cells points to the high impact of this variant on gene and protein function. According to the 2D and 3D model of *SLC20A1* protein, variant c.893T > C (p.Val298Ala, family 2) and variant c.1321A > C (p.Lys441Gln, family 3) locate both in the large intracellular loop of *SLC20A1* between TMD 7 and 8 (Figure 5A and Supplementary Data Sheet S11). Compared to variant of c.709G > A (p.Gly237Arg), the latter two variants were predicted to have less functional impact on *SLC20A1* protein function.

As outlined earlier, the BEEC incorporates a spectrum of severity, which includes the mildest form, epispadias; the intermediate form, CBE; and the most severe form, CE, also called the omphalocele, exstrophy, imperforate anus, and spinal defects (OEIS) complex (BEEC; OMIM%600057) (phenotypical pictures and more detailed description in Supplementary Data Sheet S12 (taken from Ebert et al., 2009). The BEEC is the most severe of all human CAKUT. Most affected individuals have impaired fertility despite operative reconstruction, and therefore the anomaly remains nearly always sporadic. Hence, the pathogenesis of BEEC might be explained by as yet undefined genetic *de novo* perturbations or environmental.

The SR101 assay showed high abundance of opening defects of the hindgut among *slc20a1a* MO KD zfl, resembling the imperforate anus and rectal agenesis in human CE individuals (Diamond and Jeffs, 1985). The enhanced embryonic lethality of *slc20a1a* morphants reflects the high mortality of CE, which was always fatal prior to the 1960s, when most affected individuals would die in the neonatal period (Lund and Hendren, 1993; Inouye et al., 2014). Therefore, our findings of cystic dilatations of the pronephros, cloacal disorganization, and hindgut opening defects in *slc20a1a* morphants together with the observed expression of *SLC20A1* in the urogenital sinus in a 6-week-old human embryo and the expression of *SLC20A1* in a 10-week-gestation metanephric kidney suggest *SLC20A1* to be involved in urinary tract and urorectal development.

In *slc20a1a* morphants, we saw several additional phenotypic features including growth retardation, defects of the tail and body formation, hydrocephalus, and defects in yolk sac endocytosis (Figure 1A). Accordingly, human CE presents with the exstrophic bladder, omphalocele, a rudimentary hindgut proximal to an imperforate anus, spinal defects, and intracranial anomalies comprising hydrocephalus, Chiari malformations, and craniosynostosis (Diamond and Jeffs, 1985), resembling all affected organ systems seen in *slc20a1a* morphants. While this phenotypic overlap of human CE phenotypes and *slc20a1a* morphants might be phenotypic overlap by chance, the multitude of affected overlapping organ system is suggestive of a specific effect in *slc20a1a* morphants. While our G III–IV MO KD phenotype (Figure 1A) resembles almost all if not all affected organ systems of the human CE phenotype, the milder affected *slc20a1a* MO zfl (G II) might resemble the broad phenotypic spectrum of BEEC ranging from diastasis of the symphysis only to epispadias, CBE, CE, and rare variants (Maruf et al., 2019).

Our present study supports zfl experiments for functional characterization of apparent disease genes and variants in human CAKUT phenotypes and/or congenital anorectal malformations. Additionally, our results suggest *SLC20A1* to be involved in urinary tract and urorectal development and implicate *SLC20A1* as a disease-gene for human BEEC.

## Data Availability Statement

The raw data supporting the conclusions of this article will be made available by the authors, without undue reservation, to any qualified researcher.

## Ethics Statement

The study was approved by the Ethics Committee of the Medical Faculty of the University of Bonn (No. 031/19) as well as the respective ethic committee of the collaborating centers. Written informed consent to participate in this study was provided by the participants’ legal guardian/next of kin, for the publication of any potentially identifiable images or data included in this article. Zebrafish were kept according to national law and to recommendations by Westerfield (Westerfield, 2000) in our fish facility. Written informed consent was obtained from the individual(s), and minor(s)’ legal guardian/next of kin.

## Author Contributions

HR initiated the complete study. HR and BO acquired the respective funding and provided the laboratory resources. JR and BO conceived and planned the zebrafish experiments. JR together withÖY, AJ, and MPl carried out the main experiments in zfl. DB and US planned and performed the *in vitro* experiments in HEK293 cells. FL and AW ran the IHC in human embryonic tissue. AS and TD designed the 3D model of *SLC20A1*. JR designed the figures. BO and HR supervised the work and together with JR and AW took the main lead in writing the manuscript. WN, GMB, AN, A-KE, MPr, WR, RS, KH, F-MS, ES, TB, MLa, DKl, J-HG, MA, GB, GH, GL, DKe, RC, EG, MD, WF, CM, IV, ABö, GMAB, CK, LW, PG, NZ, EJ, ABr, HT, and HR collected patients with clinical information and DNA that build the basis for the genetic analyses of this study. RZ, MLu, AH, SS, HT, and HR carried out the main analysis of the genetic data. All authors discussed the results and contributed to the final manuscript.

## Conflict of Interest

The authors declare that the research was conducted in the absence of any commercial or financial relationships that could be construed as a potential conflict of interest.

## Footnotes

1 www.graphpad.com

2 http://www.hdbr.org/

3 www.ensembl.org/

4 http://zhanglab.ccmb.med.umich.edu/I-TASSER/

5 http://zfin.org/
