# Supplementary material for: SLC20A1 Is Involved in Urinary Tract and Urorectal Development
Source: Front Cell Dev Biol. 2020 Aug 7;8:567. doi: 10.3389/fcell.2020.00567 (PMC7426641; doi:10.3389/fcell.2020.00567)
Supplement: Supplementary file 1 [file Data_Sheet_1.PDF]

Supplement 1
Figure

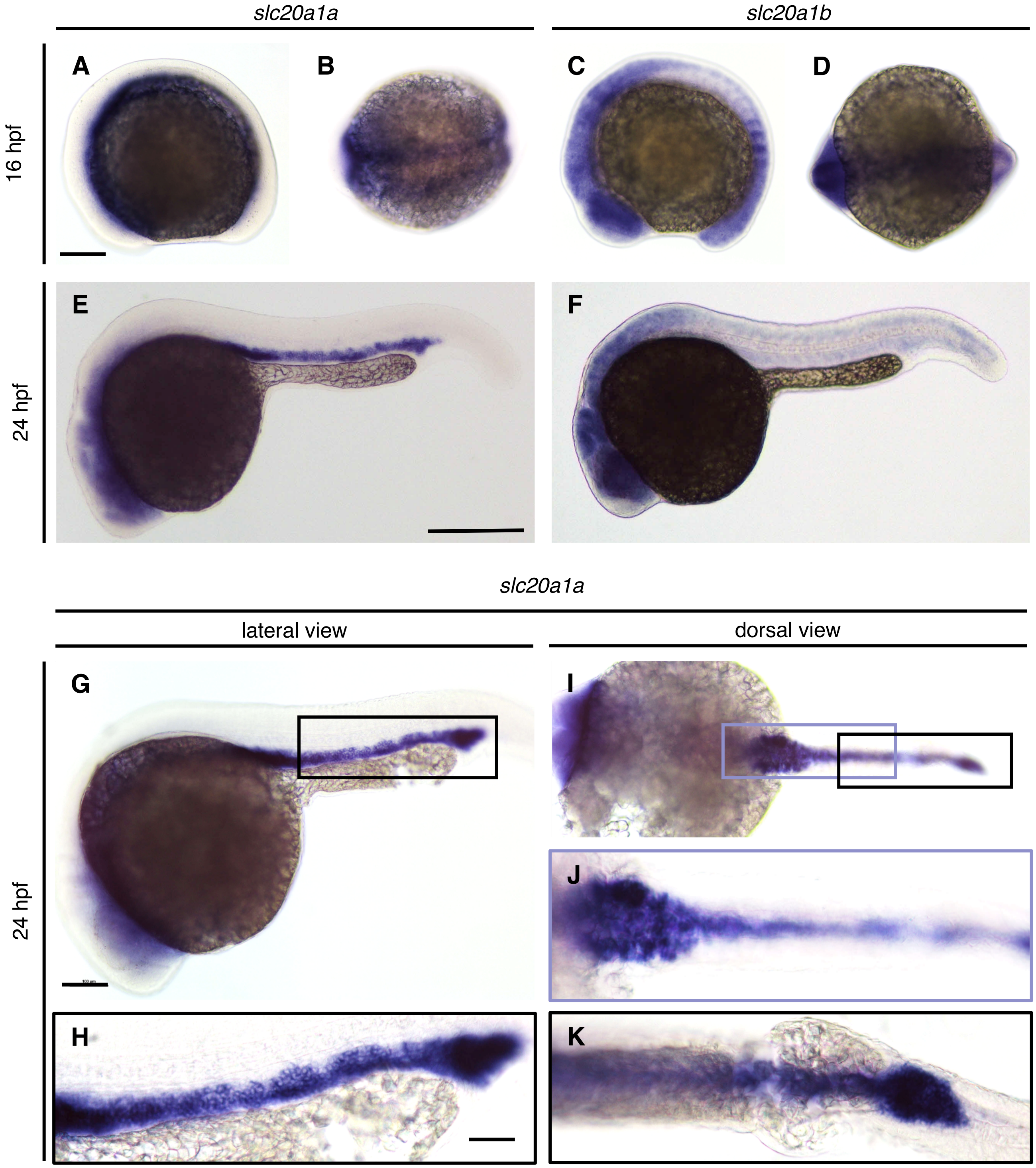

S1 Fig. Whole Mount In Situ Hybridization (WISH) against *slc20a1a* and *slc20a1b*
WISH was performed at two different time points (16 hpf: A-D; 24 hpf E-K). Expression of *slc20a1a* (A, B, E, G – K) is restricted to pronephric tissue, intermediate mesoderm and posterior cardinal vein. *slc20a1b* (C, D, F) WISH shows expression in CNS and caudal somites. There is no *slc20a1b* expression in relevant tissues for urinary tract development. Scale Bars: A-D: 100 µm; E-F: 200 µm; G + I: 50 µm; H + J + K: 50 µm
