# Supplementary material for: SLC20A1 Is Involved in Urinary Tract and Urorectal Development
Source: Front Cell Dev Biol. 2020 Aug 7;8:567. doi: 10.3389/fcell.2020.00567 (PMC7426641; doi:10.3389/fcell.2020.00567)
Supplement: Supplementary file 2 [file Data_Sheet_2.PDF]

Supplement 2
Figure

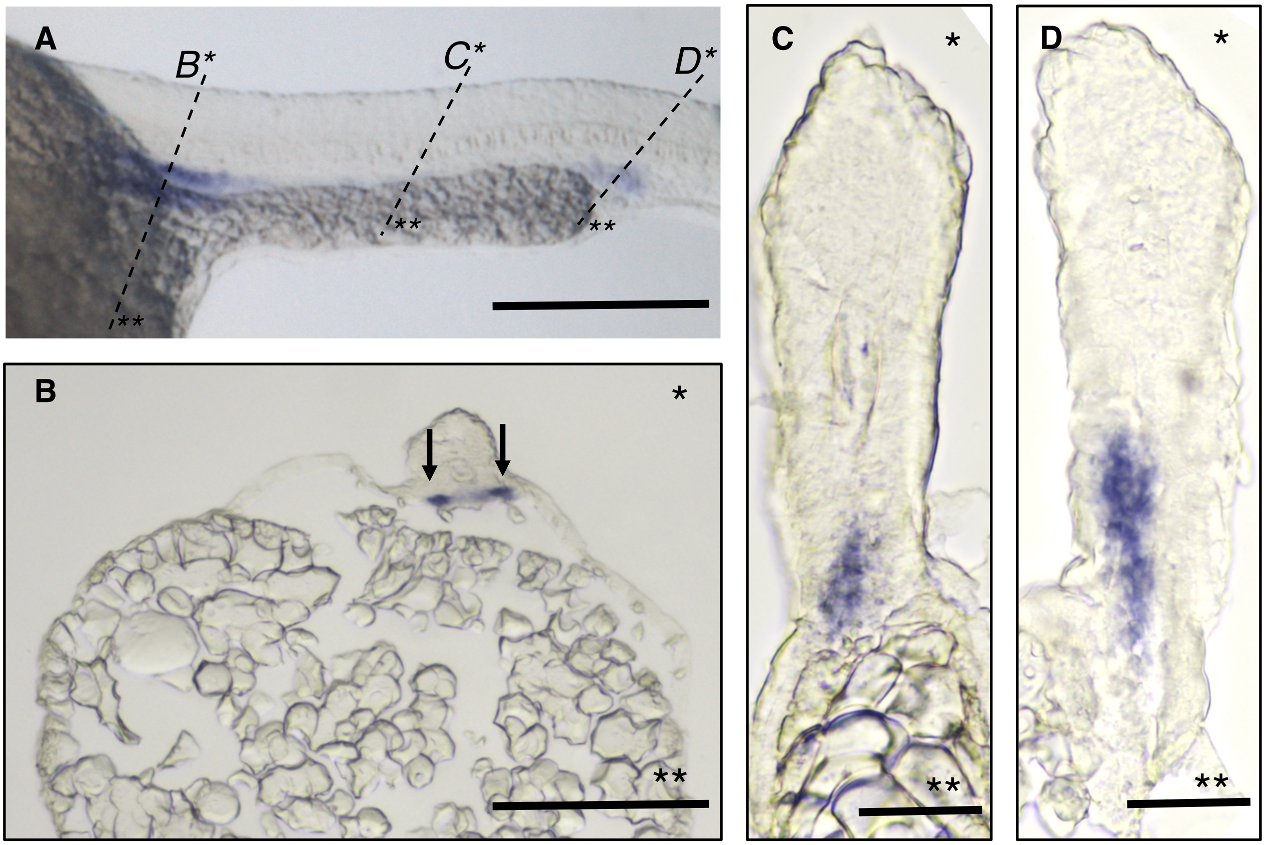

S2 Fig. Whole Mount In Situ Hybridization (WISH) against *slc20a1a,* transversal sections

WISH was performed at 24 hpf. A shows zfl in lateral view, cranial to left. B-D show transversal sections, dorsal top (*), ventral bottom (**). Dotted line in A show area of section shown in close ups B-D. Compared to S1 WISH staining reaction was stopped much earlier to demonstrate strong expression of *slc20a1a* around cloacal region at this time point of development. In B we see restricted staining to both proximal pronephric ducts (arrows). C and D show expression further caudal.

Scale Bars: A: 250 µm; B: 150 µm; C+D: 100 µm
