# Supplementary material for: SLC20A1 Is Involved in Urinary Tract and Urorectal Development
Source: Front Cell Dev Biol. 2020 Aug 7;8:567. doi: 10.3389/fcell.2020.00567 (PMC7426641; doi:10.3389/fcell.2020.00567)
Supplement: Supplementary file 3 [file Data_Sheet_3.PDF]

Supplement 3
Figure

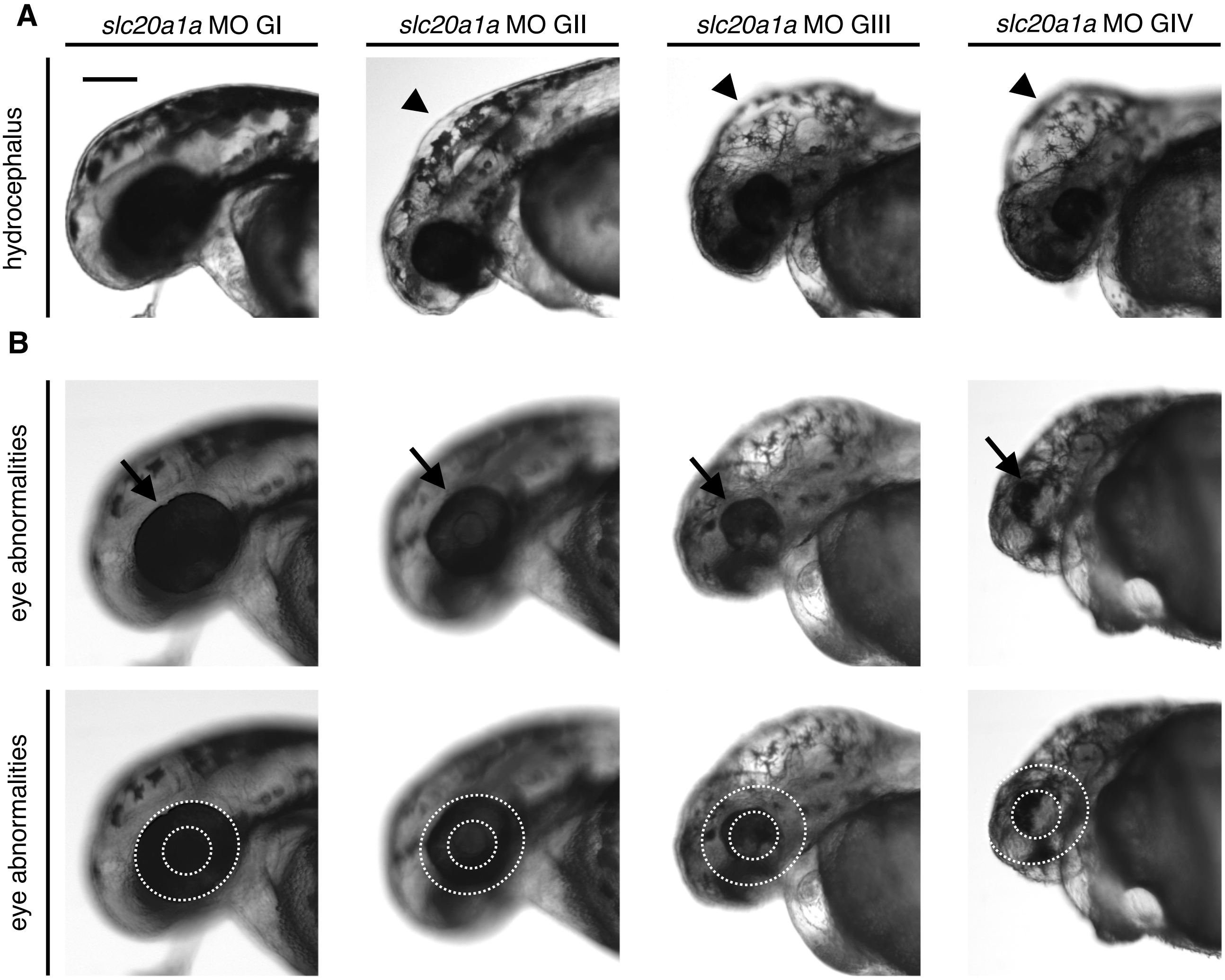

S3 Fig. *slc20a1a* MO KD Zfl show hydrocephalus and eye abnormalities increasing in severity with grading Phenotypical grading was performed at two dpf (detailed information in Figure 1). G I (no phenotype) on the left, G II second from left, G III second from right, G IV on the right. All zfl are shown cranial to the left. Scale bar: 250 µm (A) shows hydrocephalus (arrowhead), which was partially seen in G II zfl and always present in G III and G IV zfl at two dpf. (B) focuses on the left eye (arrow) and shows examples of phenotypic abnormalities seen in *slc20a1a* MO KD. G II zfl show a reduction in eye size proportional to reduction in total head and body size but no structural difference. Both, G III and G IV show severe developmental defects of the eyes of different severity. Lower panel shows eye and lens circled with white dotted lines in G I zfl. Outlines were copied from G I to G II – IV to better demonstrate reduction in size and structure (loss of lens).
