# Supplementary material for: SLC20A1 Is Involved in Urinary Tract and Urorectal Development
Source: Front Cell Dev Biol. 2020 Aug 7;8:567. doi: 10.3389/fcell.2020.00567 (PMC7426641; doi:10.3389/fcell.2020.00567)
Supplement: Supplementary file 4 [file Data_Sheet_4.PDF]

# Supplement 4
# Figure

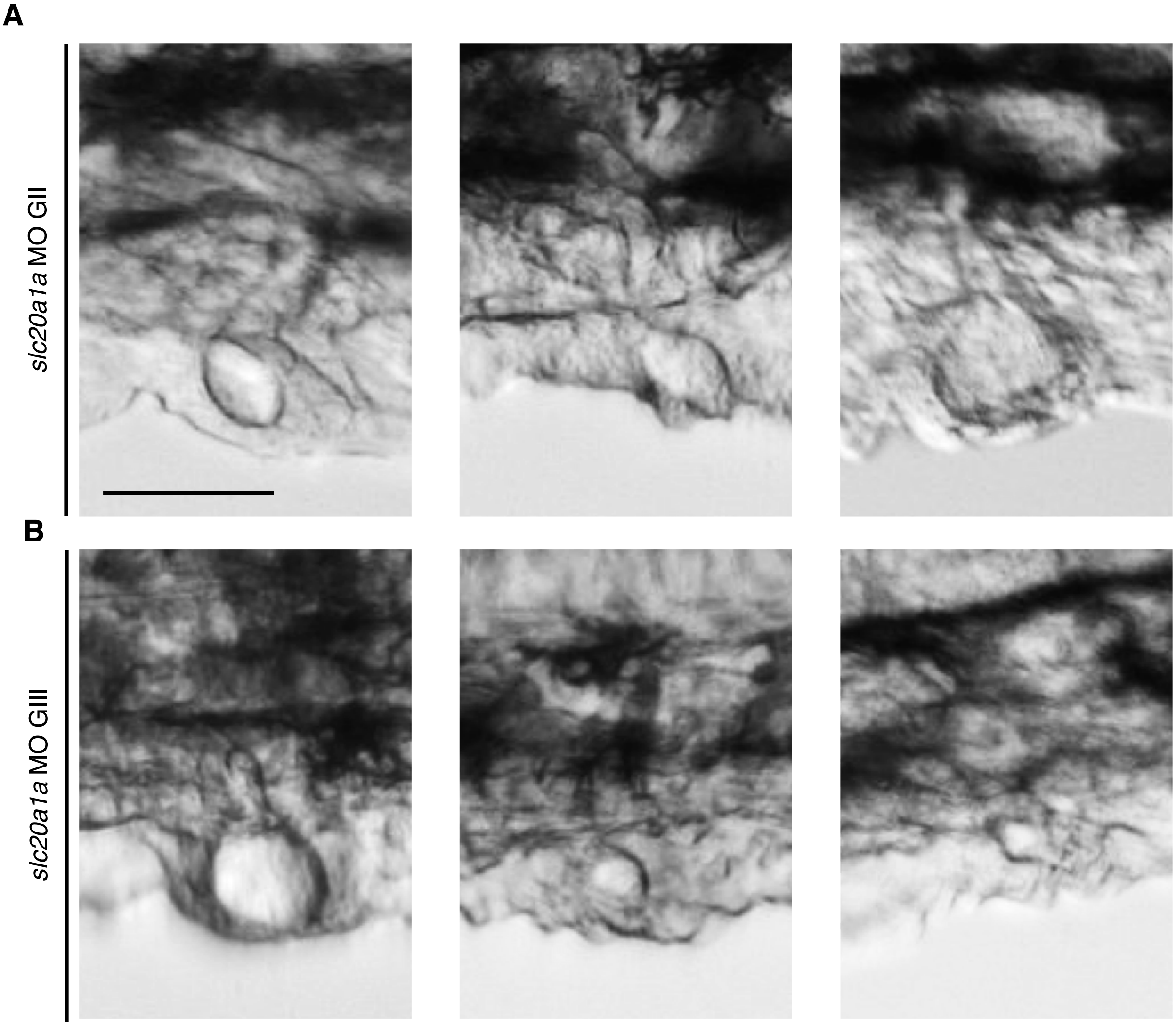


**S4 Fig. Additional examples for cloacal phenotypes in *slc20a1a* MO KD zfl at zwo dpf resembling urinary tract obstructions**

(A) shows three examples of *slc20a1a* MO KD G II zfl with high variability in shape of the urinary outflow tract. In (B) three additional phenotypes of urinary outflow tract in *slc20a1a* MO KD G III zfl are shown resembling urinary obstruction due to morphological abnormalities. Scale bar: 50 µm.
