# Supplementary material for: SLC20A1 Is Involved in Urinary Tract and Urorectal Development
Source: Front Cell Dev Biol. 2020 Aug 7;8:567. doi: 10.3389/fcell.2020.00567 (PMC7426641; doi:10.3389/fcell.2020.00567)
Supplement: Supplementary file 5 [file Data_Sheet_5.PDF]

Supplement 5 Figure

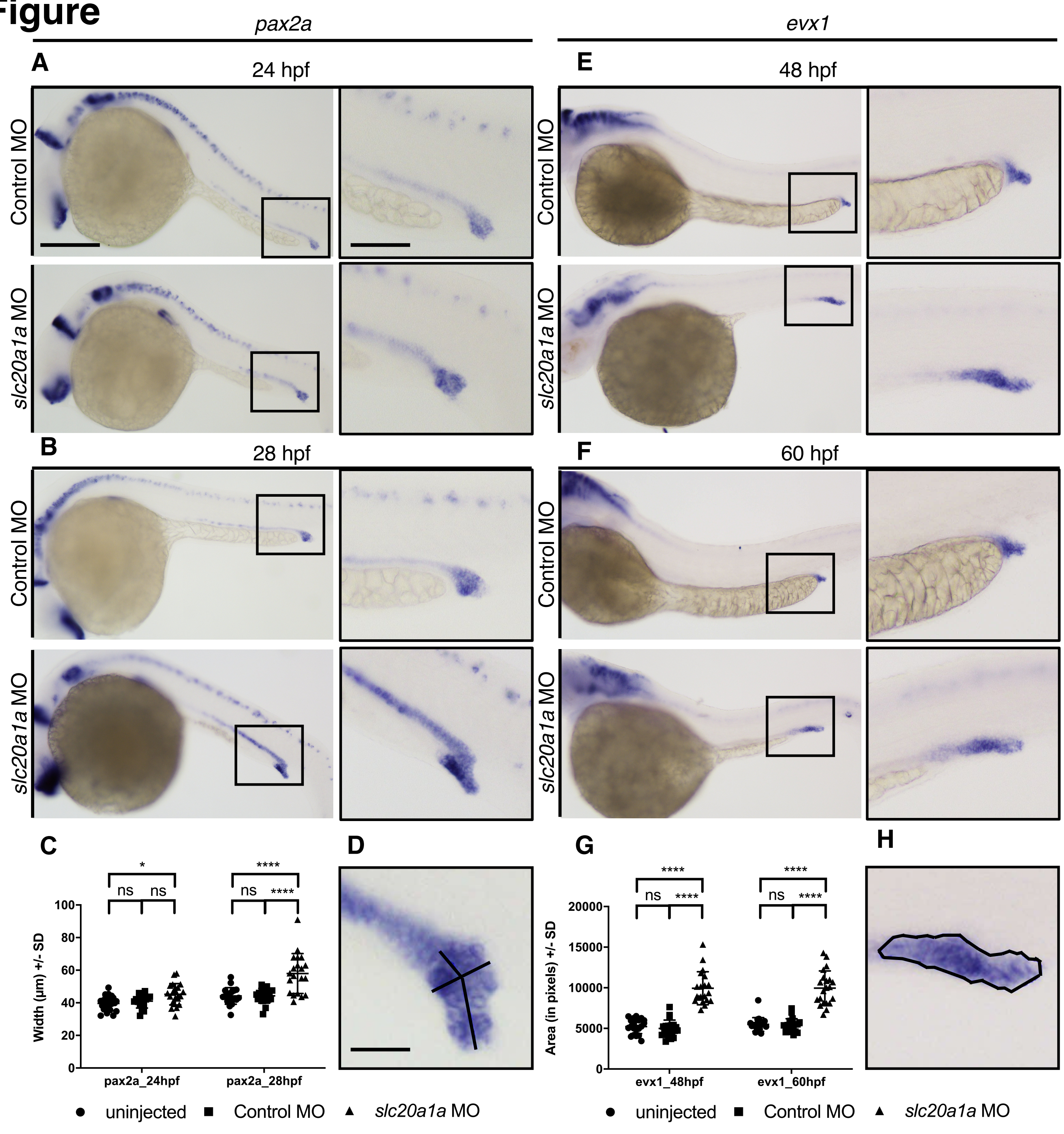

S5 Fig. WISH of marker probes in *slc20a1a* MO KD zfl compared to control MO zfl shows disorganization of cloacal tissue

Left side: WISH against *pax2a* as distal pronephric marker probe at two timepoints (A = 24 hpf, B = 28 hpf); Right side: WISH against *evx1* as cloacal marker probe at two timepoints (E = 48 hpf, F = 60 hpf). (A, B, E, F) control MO in upper panel and *slc20a1a* MO injected zfl below as well as cloacal close-ups of framed region on the right side each. Differences of expression of marker probes were seen and quantified using different approaches: In *pax2a* WISH (D) a free line was drawn in the middle of the detected region in the proctodeum resembling the urinary outflow tract at this developmental stage. The longest orthogonal to this line was measured using ImageJ. For *evx1* (H) a threshold area of expression was measured in pixels. Graphs (C, G) show mean +/- SD (Error bars) of both approaches comparing uninjected, control MO and *slc20a1a* MO zfl at both analyzed timepoints (*pax2a* in C, *evx1* in G). We see significant differences comparing *slc20a1a* MO KD with uninjected and control MO. Control MO zfl do not show any differences to uninjected group. $p < 0.05$ = *, $p < 0.0001$ = ****, hpf = hours post fertilization, Scale bars: A-B + E-F: 100 µm, D+H: 50 µm
