# Supplementary material for: SLC20A1 Is Involved in Urinary Tract and Urorectal Development
Source: Front Cell Dev Biol. 2020 Aug 7;8:567. doi: 10.3389/fcell.2020.00567 (PMC7426641; doi:10.3389/fcell.2020.00567)
Supplement: Supplementary file 6 [file Data_Sheet_6.PDF]

Supplement 6
Figure

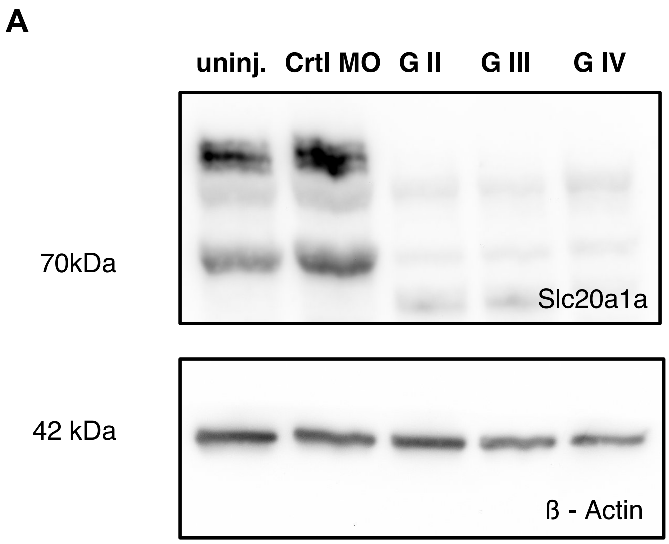

S6 Fig. Western Blot_Raw data

Raw data of Western Blot confirming Slc20a1a knockdown in *slc20a1a* MO zfl. Protein was obtained at 2 dpf. Upper panel shows WB against Slc20a1a. While there is a strong signal at 70 kDa for Slc20a1a in uninjected (uninj.) and Control MO (Crtl MO) group there is only a very weak detection of Slc20a1a at 70 kDa in *slc20a1a* MO KD groups (G II = mild phenotype, G III = moderate phenotype, G IV = severe phenotype; For detailed information on grading criteria see manuscript main text in the results section). Lower panel shows loading control ß – Actin and therefore confirms equal protein loading.
