# Supplementary material for: SLC20A1 Is Involved in Urinary Tract and Urorectal Development
Source: Front Cell Dev Biol. 2020 Aug 7;8:567. doi: 10.3389/fcell.2020.00567 (PMC7426641; doi:10.3389/fcell.2020.00567)
Supplement: Supplementary file 7 [file Data_Sheet_7.PDF]

Supplement 7
Figure

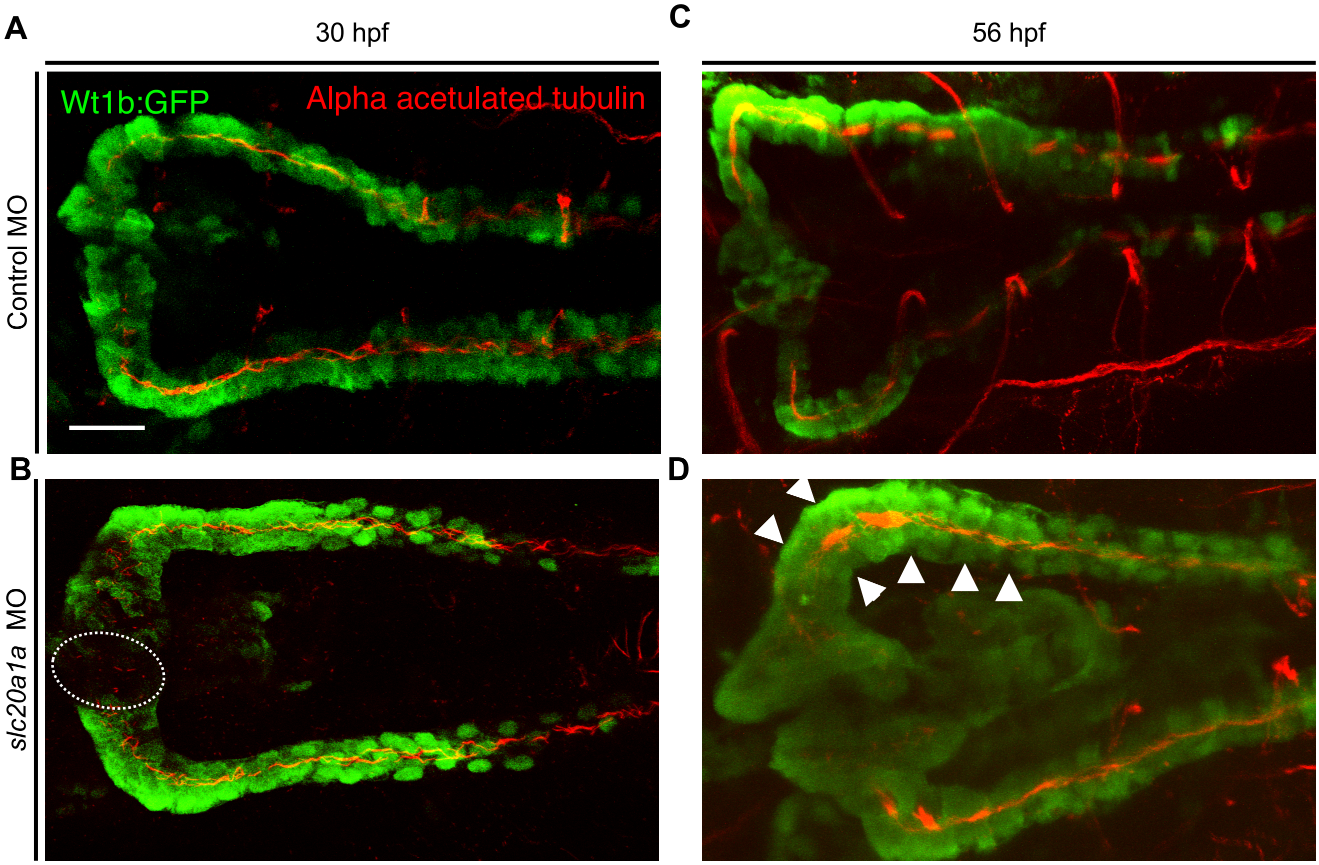

S7 Fig. Alpha-acetylated tubulin IHC staining in Tg(wt1b:GFP) showing no ciliary abnormalities but confirming pronephric dilatation and cyst formation in *slc20a1a* MO injected zfl

Control MO (upper panels, A & C) and *slc20a1a* MO (lower panels, B & D) injected Tg(wt1b:GFP) zfl at two different time points (30 hpf on left side: A & B; 56 hpf on right side: C & D) with cilia stained in red (alpha-acetylated tubulin). At 30 hpf no grading of MO KD zfl was performed due to early time point. Zfl at 56 hpf were graded and G II is shown in the images. 2-Photon imaging revealed no gross structural anomalies, such as gross shortening or elongation of cilia in *slc20a1a* MO KD zfl. Glomerular cysts in *slc20a1a* MO KD zfl were confirmed (white dotted line) and dilatation of proximal part of pronephros appeared even more prominent at 56 hpf (white arrowheads, compare to Figure 4 E). Scale bar: 50 µm. hpf = hours post fertilization.
