# Supplementary material for: SLC20A1 Is Involved in Urinary Tract and Urorectal Development
Source: Front Cell Dev Biol. 2020 Aug 7;8:567. doi: 10.3389/fcell.2020.00567 (PMC7426641; doi:10.3389/fcell.2020.00567)
Supplement: Supplementary file 8 [file Data_Sheet_8.PDF]

Supplement 8
Movie

S8 Movie. SR101 Excretion in control MO zfl

SR101 excretion assay as described before (Figure 5). Movie shows excretion of SR101 (red fluorescent dye) from the cloaca and gut peristalsis in control MO zfl at five days post fertilization.
