# Supplementary material for: SLC20A1 Is Involved in Urinary Tract and Urorectal Development
Source: Front Cell Dev Biol. 2020 Aug 7;8:567. doi: 10.3389/fcell.2020.00567 (PMC7426641; doi:10.3389/fcell.2020.00567)
Supplement: Supplementary file 9 [file Data_Sheet_9.PDF]

Supplement 9
Movie

S9 Movie: SR101 Excretion Defect in *slc20a1a* MO zfl G III

SR101 excretion assay as described before (Figure 3). Movie shows severe dilatation of distal part of gastrointestinal tract due to bag log caused by opening defects of the cloaca for the hindgut in *slc20a1a* MO G III zfl at five days post fertilization. Gut peristalsis is not reduced but clearly observable in all MO KD zfl, as seen in this movie. Scale bar: 100 µm
