# Supplementary material for: SLC20A1 Is Involved in Urinary Tract and Urorectal Development
Source: Front Cell Dev Biol. 2020 Aug 7;8:567. doi: 10.3389/fcell.2020.00567 (PMC7426641; doi:10.3389/fcell.2020.00567)
Supplement: Supplementary file 10 [file Data_Sheet_10.PDF]

Supplement 10
Table

Family	Individual (gender)	BEEC phenotype	Nucleotide Change (Transcript)<br>**Chr. Pos.** (GRCh37/hg19)	Amino Acid Change (conserved down to)<br>***Protein Domain***	ACMG (Classification of variants)	rsNumber<br>**gnomAD Allele Frequencies (hom/het/wildtype) [ethnicity, gender]**
Family 1*	I.1<br>Female	cloacal exstrophy	c.709G>A<br>(ENST00000272542.7)<br>Chr2: 113414749	p.Gly237Arg<br>(danio rerio)<br>TMD 7	Pathogenic	-
Family 2	I.1<br>female	classic bladder exstrophy	c.893T>C<br>(ENST00000272542.7)<br>Chr2: 113416516	p.Val298Ala<br>(mus musculus)<br>Intracellular loop between TMD 7 and TMD 8	Pathogenic	-<br>0.000003979<br>(0/1/251332)<br>[1 Latino female]
Family 3	I.1<br>male	classic bladder exstrophy	c.1321A>C<br>(ENST00000272542.7)<br>Chr2: 113417053	p.Lys441Gln<br>(mus musculus)<br>Intracellular loop between TMD 7 and TMD 8	Likely pathogenic	-
	II.2<br>female	division of the symphysis				

S10 Table: Heterozygous Variants of SLC20A1 in BEEC families

Family 1 reported in Reutter et al. 2016. Bladder exstrophy epispadias complex (BEEC), Chromosome Position (Chr.Pos.), homozygous (hom), heterozygous (het), Genome Aggregation Database (gnomAD), transmembrane domain (TMD), American College of Medical Genetics (ACMG)
