# Supplementary material for: SLC20A1 Is Involved in Urinary Tract and Urorectal Development
Source: Front Cell Dev Biol. 2020 Aug 7;8:567. doi: 10.3389/fcell.2020.00567 (PMC7426641; doi:10.3389/fcell.2020.00567)
Supplement: Supplementary file 11 [file Data_Sheet_11.PDF]

Supplement 11 Figure

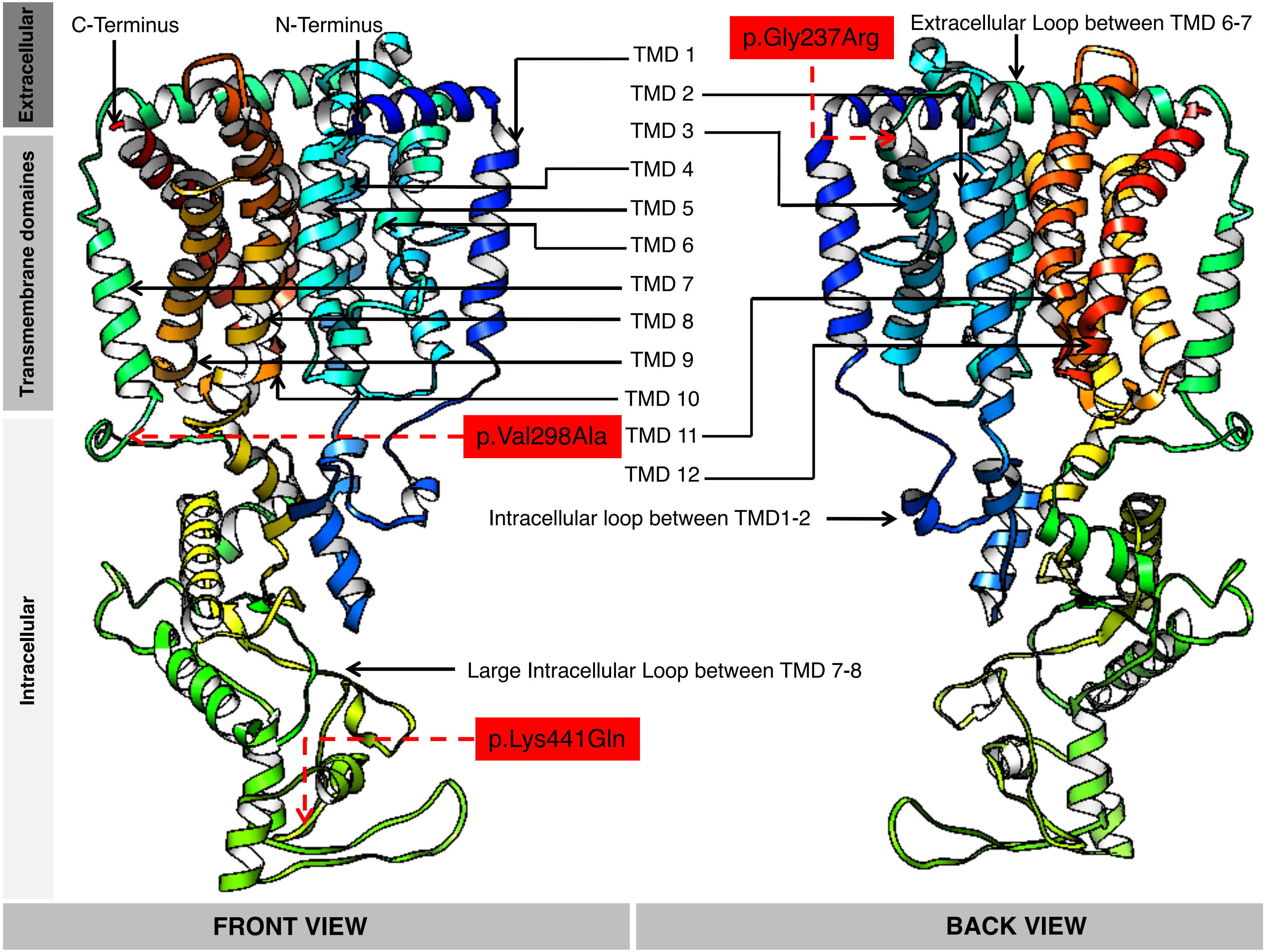

S11 Fig. Localization of variants in putative three-dimensional structure of SLC20A1 using iterative structural modeling

Caption see next page
