# Supplementary material for: SLC20A1 Is Involved in Urinary Tract and Urorectal Development
Source: Front Cell Dev Biol. 2020 Aug 7;8:567. doi: 10.3389/fcell.2020.00567 (PMC7426641; doi:10.3389/fcell.2020.00567)
Supplement: Supplementary file 12 [file Data_Sheet_12.PDF]

Supplement 12
Figure

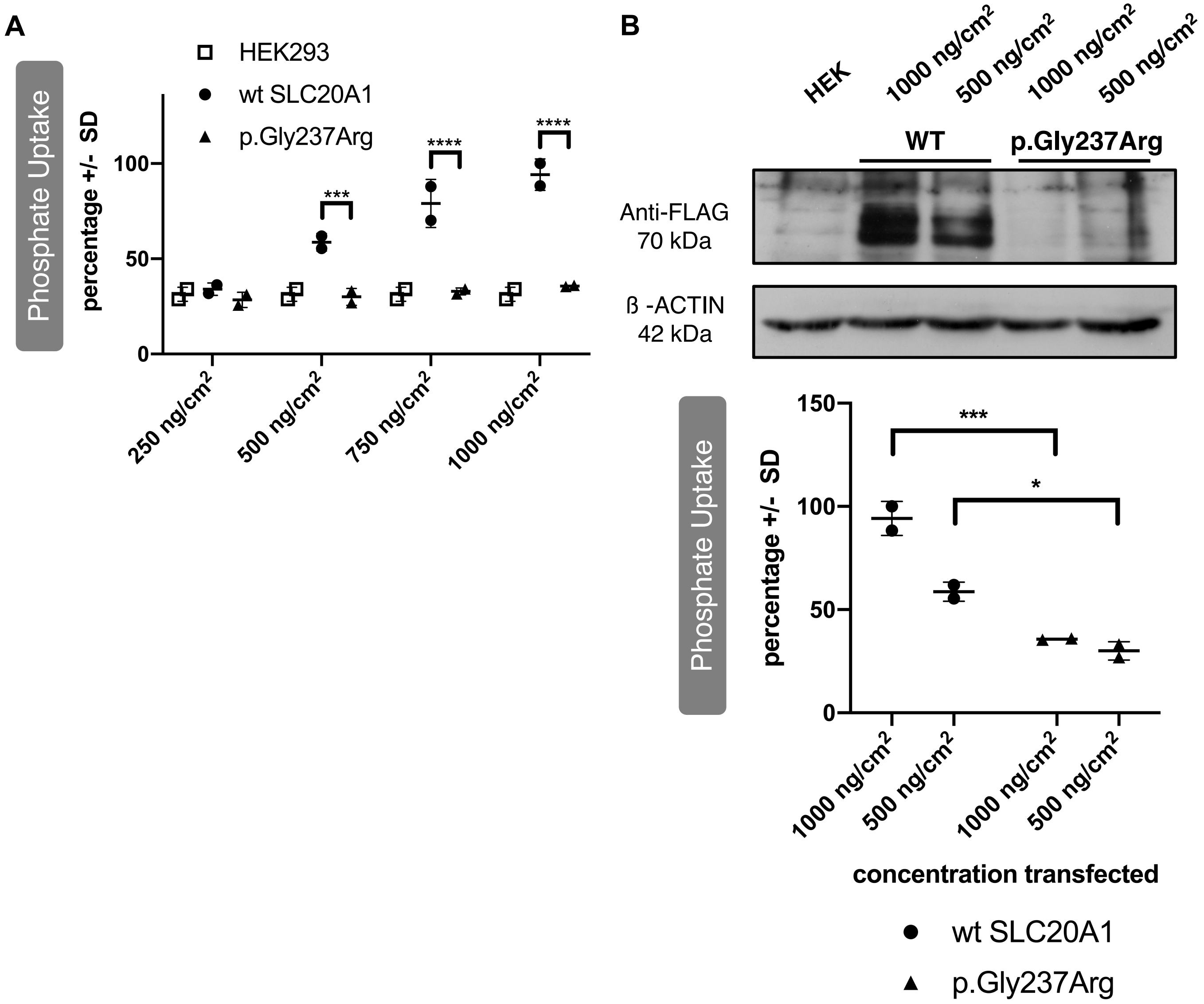

S12 Fig. Expression deficiency of c.709G>A (p.Gly237Arg) in HEK293 cells is independent of transfected plasmid concentration compared to overexpression of wt SLC20A1

Endpoint assays and WB analysis was performed as described in Figure 5B. (A) Four different plasmid concentrations (250 ng, 500 ng, 750 ng and 1000 ng plasmid DNA per cm^2 well surface) were monitored and phosphate uptake was measured. Counts per minute (cpm) were calculated in percentage with highest amount of cpm in wt SLC20A1 overexpression in highest concentration as 100 %. While phosphate uptake capability increased for wt SCL20A1 overexpression with increase of plasmid concentration transfected, no change was seen for c.709G>A (p.Gly237Arg) overexpression. Transfected plasmid amount correlates with phosphate transport in wt SLC20A1 overexpression. No phosphate transport was seen in c.709G>A (p.Gly237Arg) transfected HEK293. Error bars show SD. (B) 100 µg whole cell homogenates obtained from HEK293 cells transfected with 500 ng and 1000 ng plasmid DNA per cm^2 well surface were probed with anti-FLAG and anti-ß-ACTIN antibody. c.709G>A (p.Gly237Arg) was not expressed in HEK293 cells independent of transfected plasmid concentration. Correlation of transfected plasmid concentration and expression of wt SLC20A1 in WB was shown. For better understanding endpoint assay from A was modified showing only the corresponding values to WB. Phosphate uptake in wt SLC20A1 overexpression was significantly higher than in c.709G>A (p.Gly237Arg) overexpression for both plasmid concentrations shown (1000 ng/cm^2: $p = 0.0007$; 500 ng/cm^2: $p = 0.1$). No increase in phosphate transport was seen in c.709G>A (p.Gly237Arg) transfected HEK293 independent of plasmid concentration transfected. Error bars show SD.
