# Supplementary material for: SLC20A1 Is Involved in Urinary Tract and Urorectal Development
Source: Front Cell Dev Biol. 2020 Aug 7;8:567. doi: 10.3389/fcell.2020.00567 (PMC7426641; doi:10.3389/fcell.2020.00567)
Supplement: Supplementary file 13 [file Data_Sheet_13.PDF]

Supplement 13
Figure

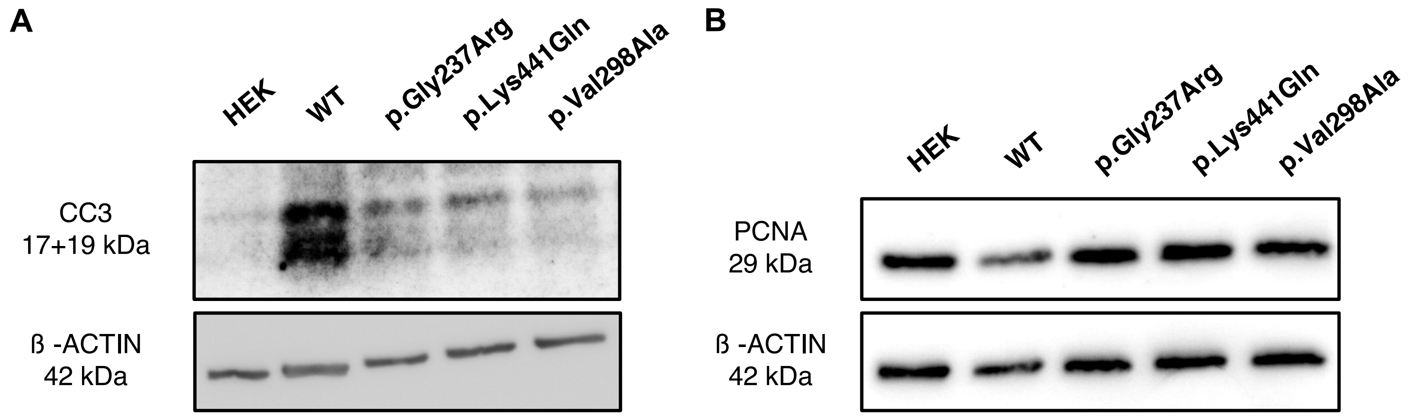

S13 Fig. Western Blot

Densitometric analysis of Western Blots (N = 6) from whole cell homogenates obtained from transfected HEK293 cells. This figure shows examples of WBs used for densitometric analysis (data presented in Figure 5D and 5E.

A shows WB against apoptosis marker CC3 and loading control beta-actin. Since CC3 ist expressed at 17 and 19 kDa both bands were used for densitometry.

B shows WB against proliferation marker PCNA and loading control beta-actin.
