# Supplementary material for: SLC20A1 Is Involved in Urinary Tract and Urorectal Development
Source: Front Cell Dev Biol. 2020 Aug 7;8:567. doi: 10.3389/fcell.2020.00567 (PMC7426641; doi:10.3389/fcell.2020.00567)
Supplement: Supplementary file 14 [file Data_Sheet_14.PDF]

Supplement 14
Figure

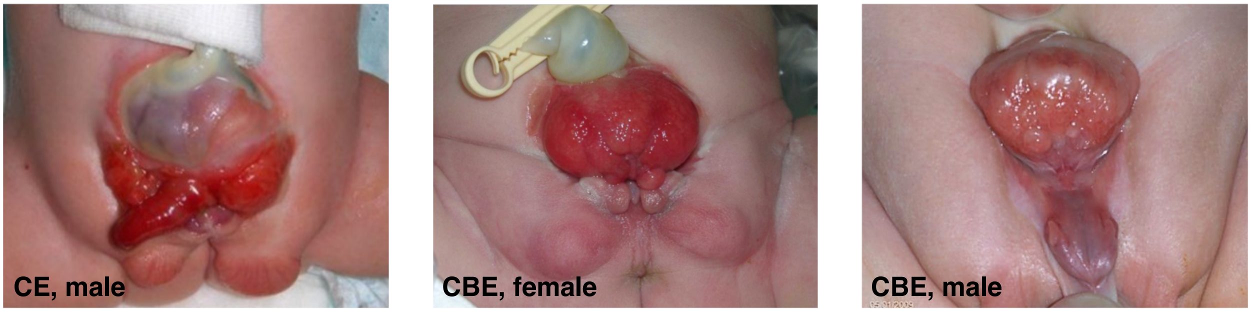

S14 Fig. Bladder Exstrophy Epispadias Complex – phenotypical spectrum

Pictures of BEEC spectrum, taken from Ebert et. al (Ebert et al., 2009). Left picture shows a male individual with cloacal exstrophy (CE) comparable to our index person (family 1) with fusion defect of pubic bone, split penis, small omphalocele and analatresia with protrusion of rectal part of intestine between unfused anteriorly exposed hemibladders. Middle picture shows classic bladder exstrophy (CBE) in an affected female individual (comparable to family 2) showing fusion defect of pubic bone, anterior exposure of dorsal wall of urinary bladder, split clitoris and forward displaced anal opening. CBE in an affected male individual (comparable to family 3) can be seen in right picture, including all features mentioned for CBE before including split penis instead of split clitoris.
