# Supplementary material for: SLC20A1 Is Involved in Urinary Tract and Urorectal Development
Source: Front Cell Dev Biol. 2020 Aug 7;8:567. doi: 10.3389/fcell.2020.00567 (PMC7426641; doi:10.3389/fcell.2020.00567)
Supplement: Supplementary file 15 [file Data_Sheet_15.pdf]

Supplement 15
Primersequences / Methods

Generation of variants for in vitro analysis

QuikChange Lightning Site-Directed Mutagenesis Kit (Agilent #210518) was used to generate variants from IMAGE clone 3918690 using following primers:

variant	forward primer	reverse primer
c.709G>A	acagaaaactgcacatctcaccgagatgaggatgg	ccatcctcatctcggtgagatgtgcagttttctgt
c.893T>C	caggccctacctcagaagcaggatgcttgttttca	tgaaaacaagcatcctgcttctgaggtagggcctg
c.1321A>C	ctccatttcttcgccctgctgttcaccttctttgg	ccaaagaaggtgaacagcagggcgaagaaatggag

Cloning for cell culture experiments

N-terminally Flag tagged wild type (WT) and mutant SLC20A1 cDNA of human origin were cloned into pcDNA3 plasmid backbone (Clontech) using the following primers:

forward:

5’ ATCTAAGCTTGCCACCATGGACTACAAAGACGATGACGACAAGATGGCAACGCTGATTACCAGTACTACAG 3’

reverse:

5’ ATCTGGGCCCTCACATTCTGAGGATGACATATC 3’;

Whole-mount Zebrafish In Situ Hybridization (WISH)

cDNA plasmids for the preparation of antisense and sense probes for pax2a, evx1, slc20a1a, and slc20a1b were generated by PCR from zebrafish poly-T embryonic cDNA using the following primers:

gene	forward primer	reverse primer
pax2a	CATCATgtcgacgcttctaacaggcacatcc	CATCATgcggccgcgatcgctatccgttcaaagc
evx1	CATCATctcgaggacaagagacagcgtctggc	CATCATggatccggtccgtgtcctgttaaaatgg
slc20a1a	CATCATctcgaggaaagctgagcgttcattgttcactg	CATCATgagctcccgaacggatggtttcgctga
slc20a1b	CATCATctcgagacctgcaaccaggatcccaaga	CATCATgagctcggagacagaacgtctgaactc

Re-sequencing of *SLC20A1* in Individuals with BEEC

Primers used for sequencing of *SLC20A1*:

name	primer sequence	name	primer sequence
SLC20A1-2F	GCTCGGTTTCTGTGCCGTAG	SLC20A1-7R	AAGCCTCCTCACCACTGCAG
SLC20A1-2R	TTATGCACAAAAATGGAACCACC	SLC20A1-8F	TACCTGCAGTGGTGAGGAGG
SLC20A1-3F	GGGTGAGCAAATTTGTGTCATGG	SLC20A1-8R	TACCTGCAGTGGTGAGGAGG
SLC20A1-4R	GGCCGCATACCTATACACCAAT	SLC20A1-9F	TTCTGTTGTGTATGTTGGGGTTC
SLC20A1-5F	ATTGGCACTATAATTCCCAAACC	SLC20A1-9R	GTCTGACCAAAATGTTTAAGGGC
SLC20A1-5R	AGCGTACACTAGCCTGCAGC	SLC20A1-10F	AGGAAACTGGGCATCTGTGG
SLC20A1-6F	GTCCCCAGAGGAGGGTTTAC	SLC20A1-10R	ACCATTTGAAAGACACACATTGG
SLC20A1-6R	TAAAAACAGTTTGGACAACAAACC	SLC20A1-11F	TCTTGTCCAGGGGTCTGGG
SLC20A1-7F	GGTGGAGGAAAGGAGACGTG	SLC20A1-11R	GGGGACCTCAGCAGGAGC
